# The reading brain – Transforming vision into language

**DOI:** 10.1016/j.pneurobio.2026.102894

**Published:** 2026-02-20

**Authors:** Avniel Singh Ghuman, Julie A. Fiez, Matthew J. Boring

**Affiliations:** a Center for Neuroscience at the University of Pittsburgh, University of Pittsburgh, Pittsburgh, PA 15213, USA; b Center for the Neural Basis of Cognition, University of Pittsburgh and Carnegie Mellon University, Pittsburgh, PA 15213, USA; c Department of Neurological Surgery, University of Pittsburgh, Pittsburgh, PA 15213, USA; d Brain Institute, University of Pittsburgh, Pittsburgh, PA 15213, USA; e Department of Psychology, University of Pittsburgh, Pittsburgh, PA 15213, USA

**Keywords:** Reading, Vision, Words, Neurodynamics, Brain connectivity

## Abstract

Reading is built upon transformations that map representations of written words to their pronunciations, names, and meanings. However, unlike many other visual skills, literacy is unique to humans and has only become widespread in the past few hundred years, therefore neural circuits that underly reading cannot have evolved for reading. Thus, scientists have long debated the nature of the neural re-tuning of visual networks that must occur to support these transformations in a highly skilled manner. A key node of the reading brain, the visual word form area (VWFA), lies where circuits that underpin key aspects of visuolinguistic transformations diverge from earlier visual processing in ventral occipitotemporal cortex. Furthermore, different reading deficits co-occur with parallel deficits in these visuolinguistic transformations. Evidence suggests that as literacy is acquired during development, preexisting visuolinguistic circuits that support representational transformations compatible with reading become tuned for understanding written words. This model implies that the VWFA is where it is because reading requires functional capacities that are parallel to those needed to recognize and name objects on one hand and those needed for multimodal integration during speech/language comprehension on the other. The VWFA is at a key location that gets input from earlier visual regions and has lateral connectivity into the auditory-visual speech and language network and ventral connectivity into visual naming circuits. We review neurophysiological, neuroanatomical, and neurodevelopmental evidence that support a model in which literacy emerges from the specialization of preexisting circuits that have the natural capacities for vision to linguistic transformations needed to acquire and facilitate fluent reading.

## 1. History and overview

Reading involves the identification of orthographic forms (letters up to words) and mapping those forms onto their name and meaning. The fact that most people hear words in our heads while we read has led to a nearly 150-year debate regarding the extent to which we represent written words in our minds at all and what the nature and neural locus of orthographic processing are. More recently, debates have emerged regarding the localization of the visual circuitry for reading, why this circuit is consistently localized across individuals, and how it becomes tuned for orthography (Dehaene and Cohen, 2007). Here we present evidence that preexisting connectivity between visual, phonological, and semantic processing regions involved in object naming and social communication are adapted to perform visuo-linguistic transformations required for skilled reading. Reading experience tunes the ventral occipitotemporal tissue located at the nexus of these pathways, which helps explain the localization and development of visual circuits for skilled reading.

Debates on the cortical localization of reading harken back to Joseph Jules Dejerine’s report in the 1890s of a case of alexia, an inability to read words holistically, instead reading letter-by-letter, without a disturbance in writing (Bub et al., 1993). This led Dejerine (and Jean-Martin Charcot) to posit that the brain has a center for the “optical images of words” in their models of how the brain processes language (Bub et al., 1993). Carl Wernicke instead argued that only letters are represented visually and are directly mapped onto their phonological forms, evident by the fact that people hear words in their heads as they read. His model had no visual center for words, only an auditory one (Damasio, 1979).

While some debate continued regarding whether sub-clinical, non-reading visual and/or naming deficits in alexic patients indicates that the disorder is not reading specific; these and subsequent studies led to the “visual word form hypothesis,” which states that there is a “visual word form system… which parses (multiple and in parallel) letter strings into ordered familiar units and categorizes these units visually.” (Warrington and Shallice, 1980)

**Fig. 1.**
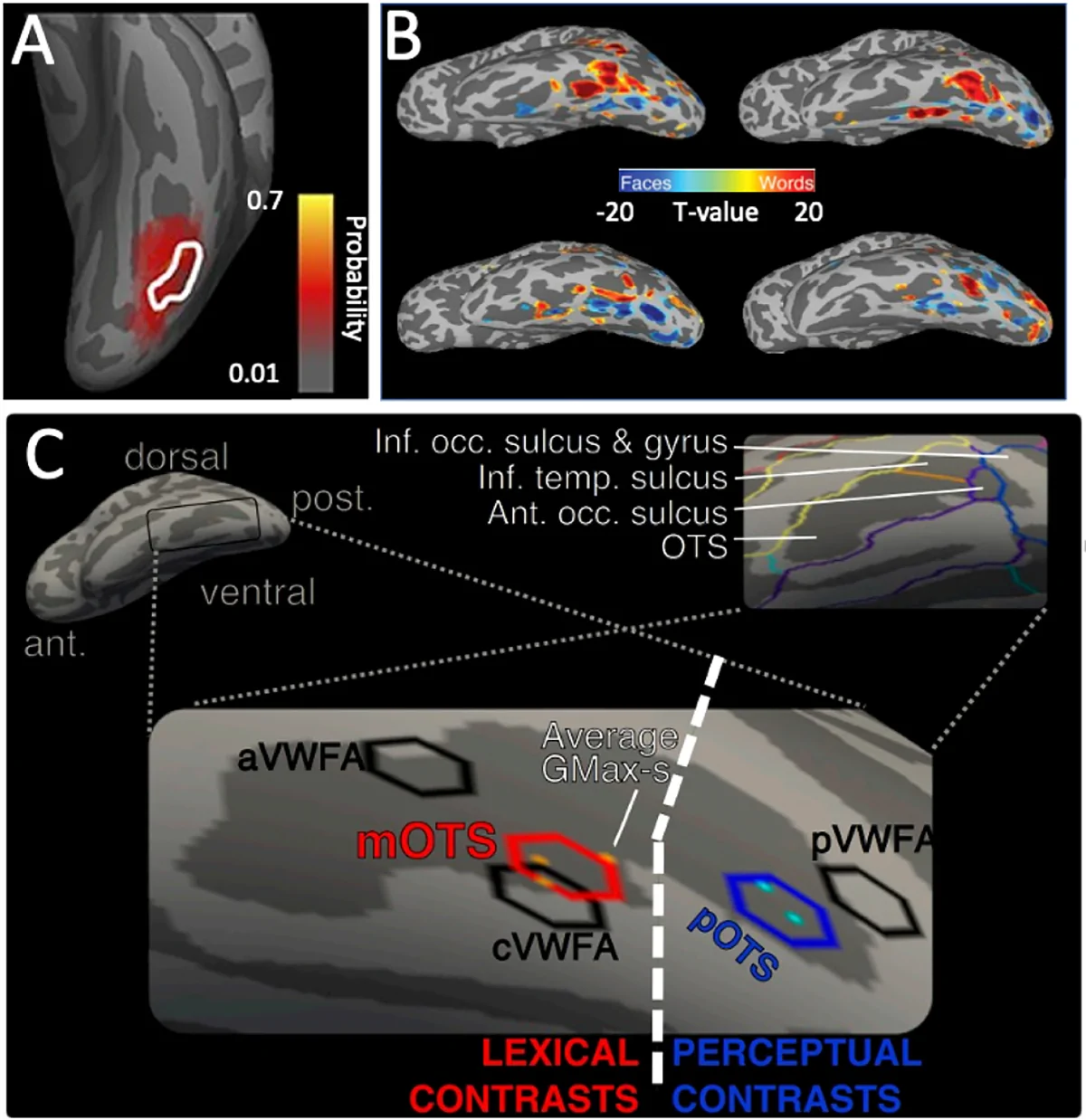
Localizing VWFA. **A)** Localization of character-selective responses in VTC. Probability that each fMRI voxel in left VTC responded to pseudowords and numbers more than faces, bodies, places and objects across 19 individuals (adapted from Rosenke et al., 2021). White outline indicates ROI demonstrating a probability of greater than 0.2 in the occipitotemporal cortex across individuals. No cortical location showed greater than ~50% probability of being character-selective, illustrating the substantial individual variability in the location of the VWFA. **B)** Variation of word-selective responses (words > faces) in four literate adult readers. Significant variability is observed across individuals, and word-selectivity is also widespread in the larger basal temporal language system (adapted from Boring et al., 2021). **C)** Functional and anatomical subdivisions have been identified in VWFA (adapted from Lerma-Usabiaga et al., 2018). Posterior occipitotemporal sulcus, sometimes referred to as posterior VWFA, has been shown to contain pre-lexical representations, whereas middle occipitotemporal sulcus (central and anterior VWFA) is selective to lexical information (i.e., at the level whole words). Spots of color (Average GMax-s) represent peak activations for different lexical (red) and sub-lexical/perceptual (blue) contrasts across subjects. OTS-occipitotemporal sulcus.

With the advent of modern neuroimaging, much of the debate around the visual word form hypothesis has revolved around a word-sensitive region in left ventral occipitotemporal cortex, sometimes referred to as the “visual word form area” (VWFA) (Fig. 1A) (Cohen et al., 2000; Mccarthy et al., 1993; Petersen et al., 1988; Rosenke et al., 2021). Here, we call this region VWFA for simplicity, although recent evidence suggests that this area contains at least two functional subdivisions (Fig. 1C) (Lerma-Usabiaga et al., 2018; Vinckier et al., 2007) and whether it may subdivide further remains an area of inquiry. There is general agreement that VWFA responds more strongly to words than other visual stimuli (Cohen et al., 2000; Mccarthy et al., 1993; Petersen et al., 1988), and damage to this region causes reading deficits (Cloppenborg et al., 2021; Hirshorn et al., 2016). However, there remains substantial debate as to whether VWFA specifically represents visual word forms, or whether this region plays a more general role in cognitive processing that reading relies heavily upon (Behrmann and Plaut, 2013; Price and Devlin, 2011, 2003), a continuation of the debate on the degree to which alexia is a “pure” deficit.

A key concept in this debate is that reading is a historically recent phenomenon; written language was invented approximately 5000 years ago, and literacy only became widespread in the past few hundred years. Therefore, it is unlikely that a visual word form system could be evolutionarily predefined for reading (Dehaene and Cohen, 2007). Instead, the circuit used for reading is purely acquired, developed, and tuned by an extended learning process. The fact that this system is not evolutionarily predefined for reading, yet VWFA is in a relatively consistent place across people, has led to debate around which neurocomputational constraints cause VWFA to become orthographically sensitive. Understanding the transformations that allow printed words to gain access to the language system may help address this debate. Most models agree that reading relies both upon mappings between orthography and phonology (e.g. sounding out printed words based on the phonology of letters and short letter strings) and lexical (whole word) mappings between orthography and whole word phonology and/or word meaning (Coltheart et al., 2001; Plaut et al., 1996; Seidenberg and McClelland, 1989).

Support for the existence of a mapping between orthography and phonology includes the fact that we can pronounce non-words/pseudowords that follow consistent orthographic-to-phonological mappings (e.g. “lerm”) but that we have not experienced before (Plaut et al., 1996; Seidenberg and McClelland, 1989). Disruption to sublexical mappings between orthography and phonology is thought to underly reading disorders like phonological dyslexia (Castles and Coltheart, 1993), where the hallmark symptom is difficulty pronouncing pseudowords while the reading of familiar words remains intact. On the other hand, there are words we can pronounce that do not follow consistent orthographic-to-phonological mappings (e.g. “yacht”). Naming of these exception words, especially those that lack orthographic neighbors that help inform their correct pronunciation (Plaut et al., 1996; Woollams et al., 2007), rely on direct or semantically-mediated mappings between orthographic representations of whole words and/or their specific pronunciations (Coltheart et al., 2001; Seidenberg, 2005). This mapping is impaired in patients with acquired surface dyslexia. One feature of this disorder is “regularization” of exception words (pronouncing “yacht” as “ya-ch-t”) (Castles and Coltheart, 1993; Seidenberg and McClelland, 1989), presumably because of dysfunctional mappings between lexical representations and their names, while sublexical orthographic-to-phonological mappings remain intact.

**Fig. 2.**
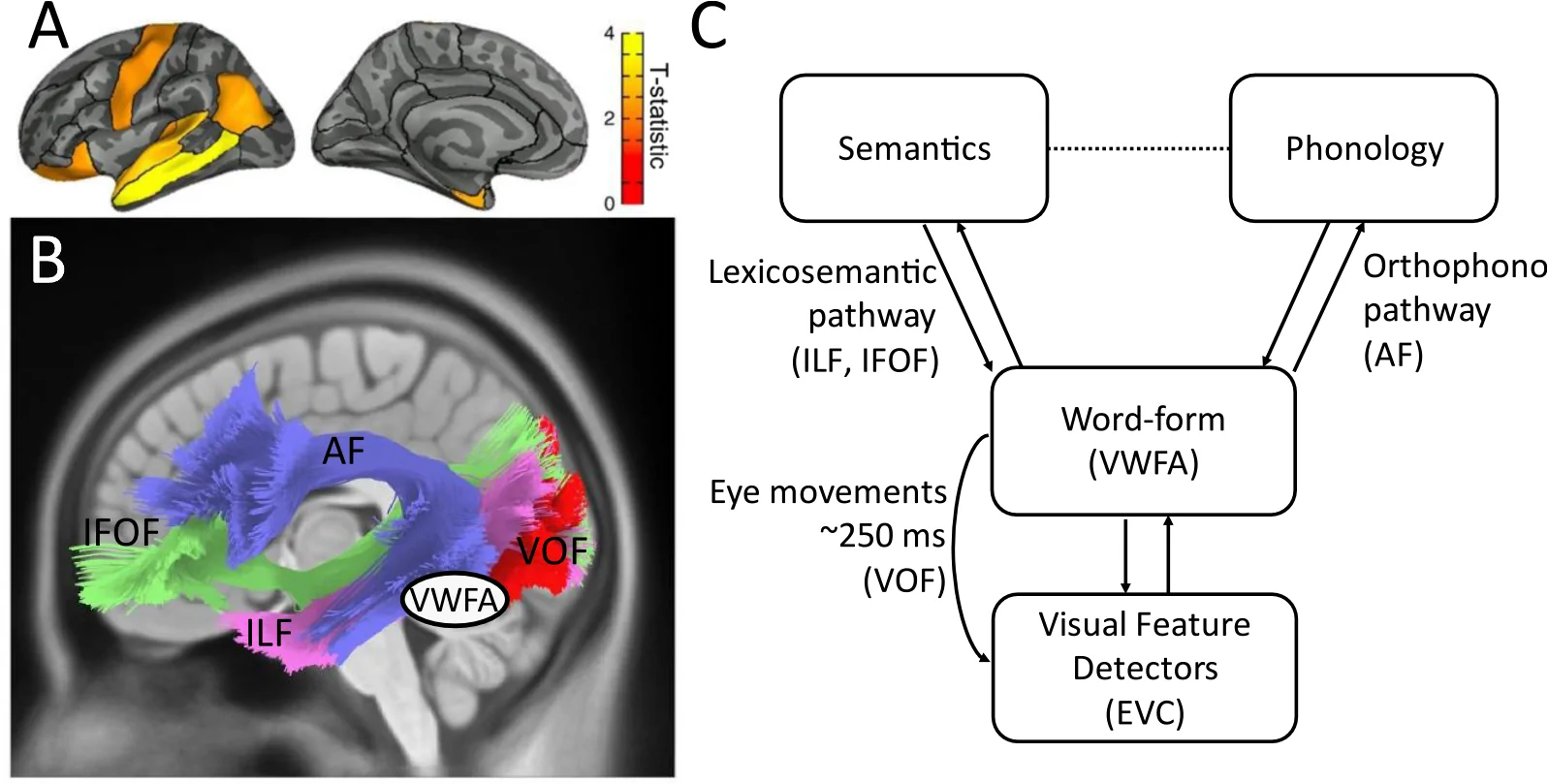
VWFA’s position in the language system. A) Difference between the strength of diffusion weighted connectivity between the region that would become VWFA and left-lateralized brain regions compared to the corresponding connectivity of adjacent face-selective cortex in preliterate five-year-olds (from Saygin et al. 2016). The region that would become VWFA demonstrated increased connectivity relative to face-selective regions to several left-lateralized language regions prior to the acquisition of literacy. Notably, these regions lie along the prominent white matter tracts shown in panel B. B) VWFA lies at a critical junction in visual, semantic, and phonological processing circuits in the brain. The arcuate fasciculus (AF, blue) links VWFA with frontal and temporal regions important for orthographic-to-phonological conversion. The inferior frontooccipital fasciculus (IFOF, green) and inferior longitudinal fasciculus (ILF, pink) connects VWFA with anterior regions of ventral temporal cortex and frontal lobe, which likely play important roles in the lexicosemantic pathway. The vertical occipital fasciculus (VOF, red) and posterior ILF connects VWFA with early visual areas important for early visual-to-orthographic mapping. In addition, the VOF connects VWFA with dorsal visual regions, which may play an important role in coordinating eye movements and visual attention during reading. This illustration was built using DSI-studio and the included ICBM 152 Atlas (Yeh et al., 2016). VWFA has been shown to be part of these pathways through fMRI functional connectivity (Saygin et al., 2016; Li et al., 2020; Stevens et al., 2017; Chen et al., 2019), anatomical connectivity (e.g., panel A; Lerma-Usabiaga et al., 2018; Li et al., 2020; Grotheer et al., 2021; Yeatman et al., 2013; Chen et al., 2019), and cortico-cortically evoked potentials (Giampiccolo and Duffau, 2022; Aron et al., 2022; Araki et al., 2015). C) Together, connectivity, neuropsychological, and neurodynamic evidence suggest a model in which low-level visual feature processors in early visual cortex (EVC) interact with VWFA to form visual representations of words. These in turn feed into and interact with higher order centers for semantics and phonology. Whether interactions between phonological and semantic centers are parallel, mutually constraining interactions for word recognition, as in Triangle models, or are serial interactions that can occur through parallel routes, as in Dual Route models (see Fig. 3), is beyond the scope of this review, hence the use of the dashed line. Interactions between visual, lexicosemantic, and phonological systems can lead to differing amounts of ambiguity in word-form representations, which may be used to program active visual information gathering (deployment of eye-movements). Abbreviations: AF-arcuate fasciculus, EVC-early visual cortex, IFOF-inferior frontooccipital fasciculus, ILF-inferior longitudinal fasciculus, VOF-vertical occipital fasciculus.

Here we provide evidence that VWFA is ideally situated at the intersection between anatomical pathways that perform transformations between visual and linguistic representations necessary for reading: bridging early visual representations of the ventral visual pathway with perisylvian, anterior and lateral temporal, and frontal language areas. VWFA is also ideally located along the ventral visual pathway for reading: it receives foveal information from earlier visual regions, providing the detailed visual information required for recognizing letters and words, while also having large enough receptive fields to integrate across letters and word parts. VWFA has connectivity into dorsal anatomical pathways appropriate for sensorimotor integration in visually-guided motor control of eye movements (Chen et al., 2019; Pan et al., 2023; Yeatman et al., 2013) and potentially writing and speech (Brownsett and Wise, 2010). This area also connects to a ventral-to-lateral temporal system (which proceeds to the perisylvian language system) that is involved in systematic visual to phonological transformation used in lip/speech reading and gesture perception, well suited for mapping orthography to its pronunciation (Saygin et al., 2016; Stevens et al., 2017). VWFA also has connectivity into a ventral-to-anterior temporal and frontal cortex pathway that maps between visual forms and their names and meaning, well suited to map whole words to their lexicosemantics (Binney et al., 2012; Hannagan et al., 2015; Saygin et al., 2016) (Fig. 2). These connections place the VWFA at an entry point for visual information to proceed into what has been called the ventral language pathway, important for lexical and semantic processing, and the dorsal language pathway, important for phonological and speech processing (Friederici, 2011; Hickok and Poeppel, 2007; Patterson and Lambon Ralph, 1999; Shekari and Nozari, 2023). Thus, this innate connectome provides access to the functional capacities and transformations that allow printed words to be processed like objects and visual communication with mappings to phonology, meaning, names, and speech. Through practice using these functional capacities, reading experience tunes the VWFA and its connectome to allow these transformations to occur with exquisite skill.

This model is an instantiation of hypotheses that large scale brain connectivity drives the specificity of local neural representations (Martin, 2006; Op de Beeck et al., 2019; Ritchie et al., 2025; Stevens et al., 2017) and bears some relation to the cultural recycling hypothesis (Dehaene and Cohen, 2007; Hannagan et al., 2015) and the neural redeployment hypothesis (Anderson, 2010). Additionally, the neurodevelopmental and connectivity concepts underpinning this model may thus provide broader insights into how acquiring perceptual expertise leverages and molds preexisting neural circuits.

Recent studies examining the acquisition of literacy and cortical responses during reading provide new insights into how learning to read adapts these pathways and begin to resolve the age-old question of whether the brain contains a visual center for whole words. Understanding the neural circuitry of reading, and VWFA’s role in this circuit, strikes at the heart of the question of the balance between nature and nurture in the brain by illustrating how functional brain architecture shapes, and is shaped by, learning and expertise (Ghuman and Fiez, 2018).

## 2. Cognitive models of reading

Two cognitive models of reading have dominated the theoretical literature on reading: the Dual Route and Triangle (Connectionist) models (Coltheart et al., 2001; Plaut et al., 1996; Seidenberg, 2005; Seidenberg and McClelland, 1989). Both were developed to account for patterns of English word naming, such as effects of word frequency and spelling-to-sound consistency on the speed and accuracy of pronouncing a word in various types of populations. Both agree that reading an alphabetic writing system (like English) involves interconnected orthographic, phonological, lexical, and semantic representations, but they differ in the nature of these connections and the constraints they provide.

**Fig. 3.**
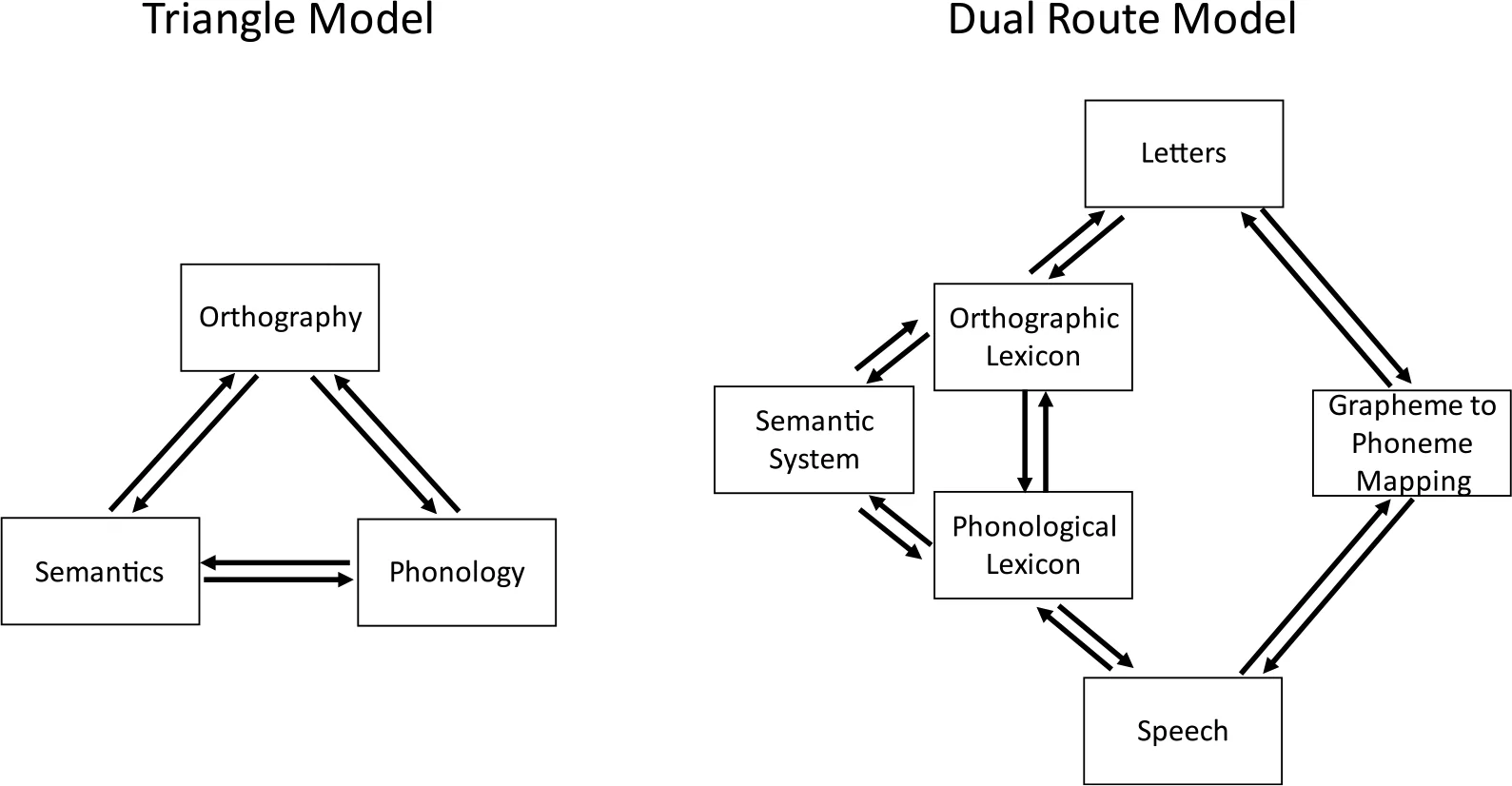
Triangle versus Dual Route models of reading. (Left) Schematic depiction of the Triangle model of reading, where learned associations between a word’s orthography, phonology, and semantics are used for reading (Plaut et al., 1996). (Right) Schematic representation of the Dual Route model of reading that contains two separate paths. The indirect path maps written words to phonology via rule-based transformations, allowing for unfamiliar words to be sounded out. The direct path maps orthography to phonology using an arbitrary mapping between orthographic representations and their specific semantic and phonological associations, which is especially important for naming exception words like yacht (Coltheart et al., 2001).

The Dual Route model characterizes reading as involving 1) a lexical orthographic-phonological and semantic pathway in which word pronunciations are retrieved by directly mapping printed words onto their whole word (lexical) level names, and 2) a sublexical orthographic-phonological pathway in which word pronunciations are assembled using rules that map graphemes (letter/letter cluster) onto their corresponding phonemes (sounds). The two routes can be used in parallel, but to pronounce an unfamiliar word or pseudoword the indirect pathway is required because a stored lexical representation is not available. To pronounce a word that violates grapheme-phoneme mapping rules (an “exception word”) the direct pathway must be used because the assembled pronunciation will yield a mispronunciation (Fig. 3) (Coltheart et al., 2001). The two routes to determining a word pronunciation are thought to reside within a larger system that includes pathways to semantic representation.

The Triangle model characterizes reading as an interactive process operationalized by parallel and distributed networks containing phonological, orthographic, and semantic information stores. Lexical-level representations are distributed across many units, and the connection weights between units are adjusted through experience reflecting joint probabilities (Fig. 3) (Plaut et al., 1996). With experience, the orthographic-phonological pathway becomes tuned to capture sublexical regularities between spelling patterns and their pronunciation, and the processing of unfamiliar printed words or pseudowords becomes weighted towards orthographic-phonological information flow. The processing of known words with statistically unusual spelling-to-sound patterns is weighted towards orthographic-semantic-phonological information flow. This means that the “division of labor” between the pathways varies across different types of items. It is the combined influence of both routes, and frequency of naming an item, that determines the final distributed pattern of activity that represents the word name (lexical phonology) (Coltheart et al., 2001; Plaut et al., 1996; Seidenberg and McClelland, 1989).

The Dual Route model characterizes phonological dyslexia as selective damage to the indirect (sublexical orthographic-to-phonological pathway), which disrupts the ability to pronounce novel words or pseudowords (Coltheart et al., 2001; Plaut et al., 1996; Seidenberg and McClelland, 1989). The Triangle model explains phonological dyslexia as damage to connections between orthographic and phonological units, disrupting the ability of individuals to generalize sublexical orthographic-to-phonological correspondences to new words (Plaut et al., 1996; Seidenberg and McClelland, 1989). The Dual Route model characterizes surface dyslexia as selective damage to the direct (lexical pathway), which disrupts the ability to retrieve the pronunciation of exception words (Coltheart et al., 2001; Plaut et al., 1996). The Triangle model explains surface dyslexia as damage to semantic units or their connections (Plaut et al., 1996; Seidenberg and McClelland, 1989; Woollams et al., 2007), which disrupts the ability to use semantic knowledge to access word names.

Despite disagreements between Dual Route and Triangle models, they both describe visual-to-orthographic, orthographic-to-phonological, and orthographic-to-semantic transformations that are part of a reading system (Coltheart et al., 2001; Plaut et al., 1996; Seidenberg and McClelland, 1989; Taylor et al., 2013). They both describe a pathway in which determining the phonological representation of a word involves sublexical orthographic mappings, and another that involves lexical orthographic mappings. We use these points of agreement as a foundation for neurobiological considerations of the reading network, recasting the pathways described in models of reading as components of an innate connectome for mapping vision into the speech-language architecture.

## 3. The organization of VWFA and its representations

Traditionally, the VWFA has been localized to the lateral part of the left mid-fusiform gyrus around (Dehaene et al., 2002). However, there is considerable individual anatomical variability, and more detailed functional parcellations of occipitotemporal cortex have demonstrated word-selectivity is observed much more diffusely around the fusiform than suggested by group level whole brain statistical maps obtained from fMRI (Fig. 1A & B). A hierarchy of word-processing regions has been identified along the length of the occipitotemporal sulcus, with more posterior aspects showing selective activation patterns for individual letters, and more anterior portions responding selectively to bigrams, trigrams (Vinckier et al., 2007), and even whole words (Forseth et al., 2018).

Another study using functional activation patterns and anatomical connectivity suggests that the VWFA comprises two subregions, a posterior prelexical region and a lexical region in the middle occipitotemporal sulcus (Fig. 1C) (Lerma-Usabiaga et al., 2018). How many subregions the VWFA contains, or whether it is a gradient of functional populations (Vinckier et al., 2007), is still under active investigation, but it is clear that the visual reading circuitry contains a number of ventral visual populations. Word-selective regions with different response properties have also been identified in middle and anterior occipitotemporal sulcus, members of an extended basal temporal language system throughout the ventral temporal lobe.

The localization of the VWFA also relates to the debate on whether orthographic representations in VWFA are sublexical, i.e. representations of combinations of letters below the level of whole words, or lexical (at the level of whole words). Studies have demonstrated prelexical representations in posterior occipitotemporal sulcus, while others (primarily focused at middle aspects of the fusiform) suggest that VWFA is lexically tuned with sublexically similar words being just as distinguishable as sublexically dissimilar words (Glezer et al., 2009).

This discrepancy is exacerbated by the poor temporal resolution of fMRI (Ghuman and Martin, 2019). Intracranial EEG supports the existence of sublexical and lexical representations by demonstrating sublexically sensitive VWFA activity prior to 250 ms after word presentation is refined to individuate orthographically similar words after 250 ms (Hirshorn et al., 2016). These more individuated representations may arise through top-down or recursive interactions that help refine the orthographic representation over time (Boring et al., 2018; Hirshorn et al., 2016; Whaley et al., 2016).

## 4. Preexisting anatomical pathways constrain VWFA’s localization

Studies in prereading children (Saygin et al., 2016) and infants (Li et al., 2020) lend support to the hypothesis that the location of VWFA is consistent across people because this part of ventral temporal cortex (VTC) has preexisting connections to broader language networks (Friederici, 2011; Hickok and Poeppel, 2007; Shekari and Nozari, 2023); however, these studies do not address why this preexisting connectivity exists. Here we argue that parallels in the visuolinguistic transformations involved in reading and evolutionarily older aspects of language and communication can explain this preexisting connectivity.

Specifically, a pathway that maps visual forms to their corresponding sounds in a consistent manner is not only core to orthographic-to-phonological transformations in reading, but also to mapping the visual forms of lips, tongue, and teeth to their corresponding consistent sounds during lip/speech reading (Campbell et al., 1986; Hannagan et al., 2015), and mapping the visual forms of limb movements to corresponding sounds during certain co-speech gesturing (particularly beat gestures) (Marstaller and Burianová, 2014). Much as there is a systematic relationship between the mouth movements/forms and sounds for syllables, there is a systematic relationship between graphemes and phonemes. On the other hand, a direct, non-systematic/arbitrary mapping from visual forms to words is seen not only for exception word reading, but also for object, place, and person names (Brédart, 2017). These similar transformations for reading and other visuolinguistic processing may help explain why face-, limb-, object-, and word-selective areas are close together in VTC (Grill-Spector and Weiner, 2014; Hannagan et al., 2015; Nordt et al., 2021; Op de Beeck et al., 2019).

The model we lay out here is that these pathways are a “connectomic fingerprint” of what becomes the VWFA, likely present at or before birth, and are present due to evolutionary pressure for visual processing pathways related to speech perception and naming that are important for communication and survival. The process of acquiring literacy leverages the functional capacities of this connectomic fingerprint. Through extensive reading practice and innate mechanisms for neuroplasticity, the VTC and its connectome are tuned to allow for the fluent mapping of visual word forms to their corresponding phonology and meaning.

## 5. The anatomy of the early visual-to-orthographic pathway

The vertical occipital fasciculus and anterior aspects of the inferior longitudinal fasciculus connect retinotopic striate visual cortex to VWFA and the other parts of higher level visual processing areas in VTC. Numerous studies have demonstrated that retinotopic biases persist all the way up the visual processing stream (Groen et al., 2022; Wandell et al., 2007; Wang et al., 2015), likely arising from differential connectivity between foveal and peripheral processing aspects of early visual cortex and lateral (foveal preference) and ventral (peripheral preference) VTC. These results have led to the hypothesis that the organization of category preference in VTC at least in part reflects where particular types of visual stimuli typically fall in the visual field (Groen et al., 2022). Specifically, visual categories that are typically foveated, such as faces and words, tend to be clustered in more lateral aspects of VTC connected to foveal processing parts of early visual cortex and categories that tend to fall in the periphery, such as navigational and scene information, tend to be clustered in more medial aspects of VTC connected to peripheral processing parts of early visual cortex (Hasson et al., 2002). VWFA falls within lateral VTC that has foveal preference (Hasson et al., 2002) and is connected to foveal processing parts of earlier visual cortex.

## 6. The anatomy of a dorsal visuomotor and attention pathway in reading

**Fig. 4.**
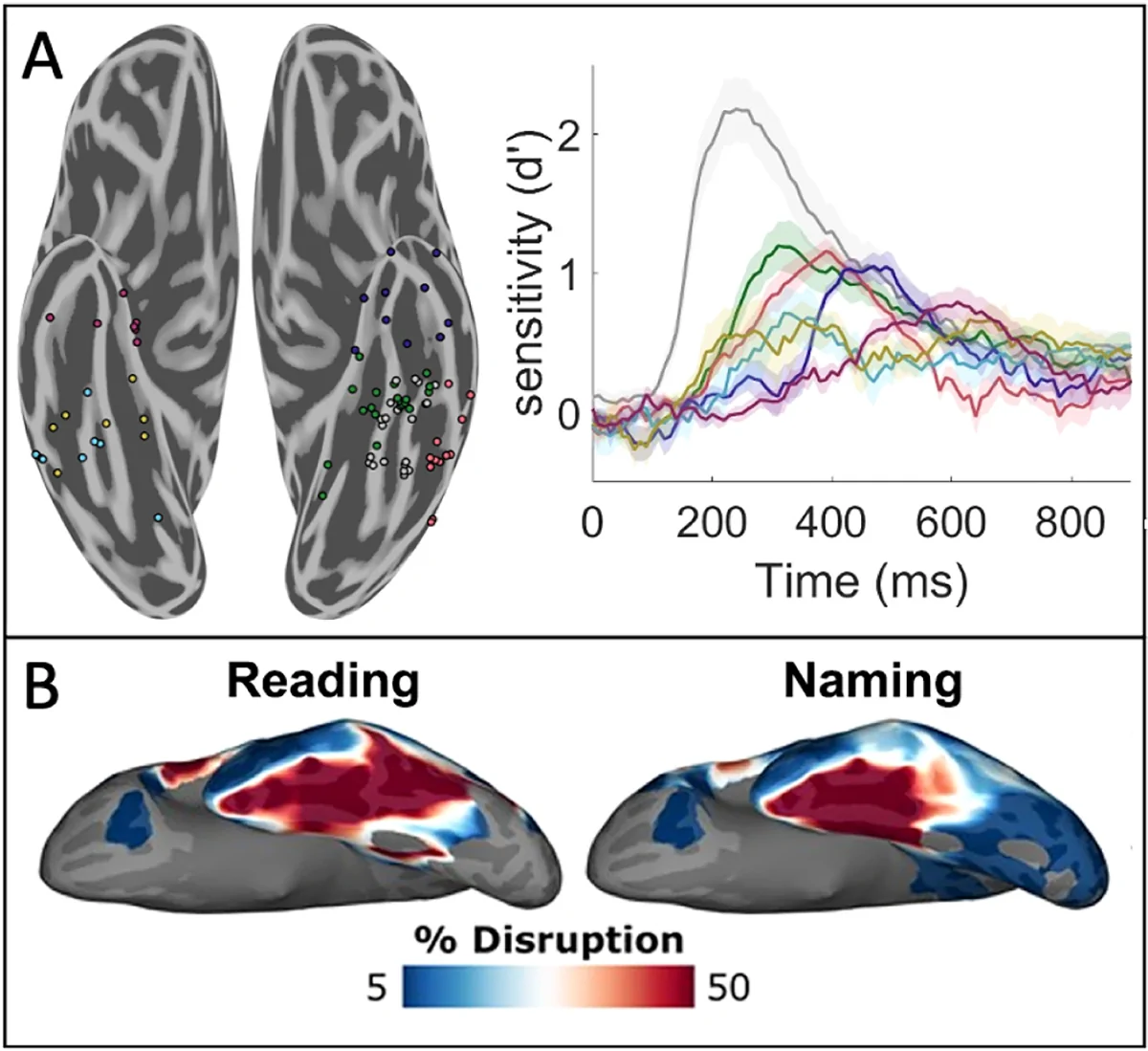
The dynamics and function of the basal temporal language system. **A)** (Left) Ventral surface of inflated brain. Dots represent word-selective intracranial electrode contacts across 36 patients (taken from Boring et al., 2021). These contacts were clustered based on their spatial location and the time-course of their word-selective responses. Color represents the spatiotemporal cluster they belong to, the average time-course of which are illustrated in the plot to the right. Word-selective contacts are found throughout VTC. VWFA likely corresponds to the grey colored contacts based both on their anatomical location and their early response. The other electrode clusters correspond to putative subregions of the BTLS. (Right) Average time-course of word-selective responses recorded from intracranial contacts plotted on the brain to the left. Word-selective responses are strongest and earliest (peaking at ~225 ms) along the left posterior fusiform gyrus (VWFA), before spreading medially and laterally from there. Finally, anterior word-selective responses peak around 400 ms after word presentation. Right hemisphere word-selective VTC responses did not peak until approximately 400 ms. **B)** Electrical brain stimulation across left VTC of 49 patients showing both the overlap between reading and naming in BTLS and the dissociation between reading and visual object naming in VWFA, adapted from Woolnough and Tandon (2024). Both reading and naming are disrupted during electrical stimulation to large swaths of anterior and medial VTC, the BTLS, which thus participates in lexicosemantic processing. Conversely, stimulation to the VWFA, which is in mid-lateral VTC, only disrupts reading.

Reading text is a voluntary motor act that is guided by visual input and often involves coordinated speech planning and production. The visual guidance needed to provide motor control of reading is likely provided by the vertical occipital fasciculus, which loops vertically to the dorsal visual and parietal regions involved in visually-guided attention and movement planning (Komatsu and Wurtz, 1988). During natural reading, we move our eyes approximately 3–5 times per second (Rayner, 2009), with the timing and locations of fixation tightly associated with the timing of phonological access and visual word identification. It has been suggested that this connectivity between posterior VWFA and inferior parietal regions may be critical for eye movement guidance during reading (Chen et al., 2019; Pan et al., 2023; Yeatman et al., 2013). Notably, word selectivity of left hemisphere VWFA peaks between 200 and 250 ms (Fig. 4A; (Boring et al., 2021), a timing well suited to contribute to eye movement guidance given the frequency of saccades.

The vertical occipital fasciculus pathway may also be important for the intentional coordination of gaze fixation and reading-related speech (such overtly or covertly sounding out a word based on explicit grapheme-phoneme knowledge(Alvarez and Fiez, 2018)). The pathway includes projections to a region of the intraparietal sulcus involved in visually guided orofacial movements, and from this orofacial region there are projections to perisylvian regions associated with speech planning and production (Brownsett and Wise, 2010). Additionally, this projection may be critical for visual-motor integration for writing and endpoints of the vertical occipital fasciculus in the parietal cortex/temporo-parietal junction have been implicated in writing as well (Brownsett and Wise, 2010).

Many open questions remain regarding the neurobiology of visuomotor integration during natural reading, including consideration of the different types of reading eye movements, such as regressive saccades. Furthermore, the role of visually-guided speech planning and production particularly in beginning reading, when most printed words are unfamiliar and “sounding out” their pronunciation is an effortful process, remains to be fully explored. While current findings are relatively circumstantial, taken together they suggest that connections along the vertical occipital fasciculus may be key to saccade and speech programming during natural reading. Further, through connecting pathways into the frontal lobe, parietal regions involved in eye-speech coordination have access to frontal regions with connections to the VWFA. This raises the possibility that the dorsal motor control pathway has an important role in attentional processes that influence orthographic learning in the VWFA.

## 7. The anatomy of the orthographic-to-phonological pathway

Functional and anatomical connectivity studies implicate connections between VWFA and perisylvian language regions involved in phonological processing as a key pathway for orthographic-to-phonological mapping (Grotheer et al., 2021; Gullick and Booth, 2015; Vandermosten et al., 2012a; Yeatman et al., 2011), likely via the posterior arcuate fasciculus that has terminations around anterior VWFA (Fig. 2) (Bouhali et al., 2014; Giampiccolo and Duffau, 2022; Lerma-Usabiaga et al., 2018). The VWFA connects dorsally to phonological processing regions in the superior temporal cortex and neighboring parietal cortex (Saygin et al., 2016; Stevens et al., 2017; Yablonski et al., 2024) and further on into pre- and perimotor speech-language regions involved in articulatory planning and subvocalization during reading via the arcuate fasciculus (Friederici, 2011; Hickok and Poeppel, 2007; Shekari and Nozari, 2023). Note that we use “perisylvian language areas” loosely to refer to the phonological and articulatory processing areas served by the arcuate fasciculus and thus includes frontal areas somewhat more dorsal than are typically described as perisylvian that have sometimes been implicated in phonological and subvocal articulatory processing in reading (Tomasino et al., 2020; Woolnough et al., 2022). This pathway is especially important for pseudoword reading (Hoffman et al., 2015; Richlan et al., 2009; Vandermosten et al., 2012b), which leverage sublexical orthographic-to-phonological mapping. Furthermore, aberrant activity in these regions and abnormal connectivity in this pathway are associated with phonological dyslexia (Silani et al., 2005; Vandermosten et al., 2012a).

Our brains are not innately wired for reading, but they are wired for multimodal sensory integration and auditory word learning. When listening to others, we often receive a simultaneous stream of visual and acoustic speech and much as there is grapheme-to-phoneme correspondence, there are chunks of visual information from visual face, mouth, and lips that correspond to syllables. Thus, the orthographic-to-phonological pathway from the VWFA dorsally into the perisylvian language system may leverage this multimodal visual-speech integration pathway. Indeed, a few studies implicate a putatively similar pathway for integrating mouth forms with their sounds (lip/speech reading) (Erickson et al., 2014; Michon et al., 2019), though direct evidence for co-localization of lip/speech reading and word reading pathways within subjects is lacking (Hannagan et al., 2015). In particular, regions of mid-to-anterior superior temporal sulcus and frontoparietal motor and premotor regions that show connectivity to VWFA prior to learning to read (Fig. 2A) are similar to the regions implicated in vocal audio-visual integration (Calvert et al., 1997; Hauswald et al., 2018) and the McGurk effect (Erickson et al., 2014). A recent study demonstrated a moderate-to-large statistical relationship between the magnitude of the McGurk effect and reading decoding and comprehension (Pulliam et al., 2025), supporting the hypothesized shared pathway for lip/speech reading and word reading. Furthermore, individuals with phonological dyslexia demonstrate co-occurring deficits in lip/speech reading (De Gelder and Vroomen, 1998; van Laarhoven et al., 2018) and do not benefit as much as non-dyslexic individuals from viewing speakers while listening to their speech in noise (Francisco et al., 2018). Although poor phonological representations could impact both lip/speech reading and word reading ability without the two sharing a common pathway, neuropsychological studies provide evidence that a single lesion can cause both lip/speech reading and word reading deficits (Albonico and Barton, 2017; Campbell et al., 1986). In one of these studies (Campbell et al., 1986), two patients had strokes in an artery that caused damage to VTC. The patient with damage to the left hemisphere had alexia and deficits in lip/speech reading ability, despite no signs of prosopagnosia, while the patient with right hemisphere damage had prosopagnosia but had intact lip/speech reading ability.

The relative paucity of empirical testing of the overlap between lip/speech reading and word reading in the brain is a significant limitation to understanding the orthographic-to-phonological pathway. Specifically, it remains relatively untested as to whether VWFA contributes to differentiating mouth forms. Furthermore, it is unclear if regions involved in auditory-visual integration in speech play a role in orthographic-to-phonological transformations and lexical phonology in reading. Functional and anatomical mapping studies of word and lip/speech reading within the same subjects, particularly in children learning to read, are required to assess whether these processes use joint or non-overlapping, but parallel, pathways.

## 8. The anatomy of the orthographic-to-lexicosemantic pathway

Neurological, intracranial electrophysiology, and neuroimaging studies suggest that a pathway through medial and anterior VTC via the inferior longitudinal fasciculus and/or inferior frontal occipital fasciculus (Fig. 2), that parallels the object naming pathway, is a key part of the lexicosemantic pathway (Forseth et al., 2018; Hermann et al., 1999; Liuzzi et al., 2019; Lüders et al., 1991; Ueno et al., 2018). This pathway connects the VWFA anteriorly along VTC through the temporal pole and into areas of the inferior frontal cortex that have been implicated in lexicosemantics during reading, naming, and perceptual recognition (Forseth et al., 2018; Ghuman et al., 2008; Graves et al., 2007; Luders et al., 1991; Mesulam et al., 2013). The neurological literature has long appreciated that stimulation of many places along VTC can cause speech and language dysfunction and has termed this area the “basal temporal language area.” This “area” has been defined as spanning from 3 to 9 cm from the tip of the temporal lobe (Lüders et al., 1991) and therefore likely consists of multiple areas along a basal temporal language system (BTLS). Indeed, recent studies using intracranial recordings and stimulation have highlighted that BTLS consists of multiple areas contributing to multiple, temporally extended aspects of reading and language (Boring et al., 2021; Forseth et al., 2018; Woolnough et al., 2021) (Fig. 4). Cortico-cortically evoked potentials have demonstrated connectivity between eloquent regions of basal temporal cortex (Aron et al., 2022) and between these regions and perisylvian language areas (Araki et al., 2015; Koubeissi et al., 2012). Furthermore, an area that shows connectivity to what becomes VWFA prior to learning to read is in anterior BTLS (Fig. 2A; Saygin et al., 2016), suggesting that connectivity to BTLS helps constrain the location of VWFA once literacy is acquired.

Neuroimaging atlases of reading often neglect BTLS regions medial and anterior of VWFA, perhaps due to susceptibility artifacts in anterior temporal cortex (Devlin et al., 2000) or differential sensitivity of the fMRI signal to different aspects of the electrophysiological response (Ghuman and Martin, 2019; Jacques et al., 2016). Notably, a few studies of reading that used fMRI techniques designed to increase signal-to-noise ratio in VTC have associated the extended BTLS with reading (Boring et al., 2021; Hoffman et al., 2015). Evidence suggests that the medial-to-anterior BTLS plays a critical role in the lexicosemantic pathway, particularly in some aspects of exception word processing (Hoffman et al., 2015; Wilson et al., 2012; Woolnough et al., 2021). Damage to BTLS and the inferior longitudinal fasciculus is associated with surface dyslexia (Tomasino et al., 2020, 2015). Additionally, transcranial magnetic stimulation of left anterior BTLS causes regularization of exception words (Ueno et al., 2018) (e.g. temporary surface dyslexia) and a consistent early symptom of anterior temporal lobe degeneration in semantic dementia (also called “the semantic variant of primary progressive aphasia”) is surface dyslexia (Hodges et al., 1992).

One point to note is that, though BTLS has a substantial left hemisphere bias, aspects of the BTLS appears to be more bilaterally organized compared to the stricter left lateralization of much of the speech-language system. Resections of left BTLS for the treatment of epilepsy manifest with mild and inconsistent naming and word finding deficits (Rice et al., 2018), though larger resections of BTLS cause naming deficits more consistently (Kaestner et al., 2022; Snyder et al., 2023). Bilateral BTLS degeneration associated with semantic dementia causes consistent and more severe naming deficits (Binney et al., 2016; Mesulam et al., 2013). Furthermore, both left and right BTLS display word selectivity at 300–400 ms after word presentation, with left VWFA becoming word selective about 100 ms earlier (Boring et al., 2021) (Fig. 4A). One potential lateral asymmetry of BTLS may be between naming and semantic processing, where left BTLS is more important for naming than right BTLS, but broader semantic processing is distributed in a more distributed and bilateral fashion. Disentangling this lateralization requires examining the effects of left and right BTLS stimulation and damage on naming, verbal and non-verbal tests of semantic knowledge. The bilaterality of BTLS may help explain why non-alphabetic writing systems (like the one used in written Chinese), which may rely more heavily on orthographic to lexicosemantic mappings than alphabetic systems, display stronger bilateral activation compared to alphabetic writing systems (Yang et al., 2006).

VTC in the proximity of BTLS also plays a critical role in naming objects, people, and places (Bédos Ulvin et al., 2017), which, like exception words, cannot be named using consistent visual to phonological mappings (Brédart, 2017). Thus, the lexicosemantic pathway for written word naming may be parallel to, and/or overlapping with, the broader visual naming pathway. Much as phonological dyslexia and lip-reading deficits co-occur (De Gelder and Vroomen, 1998; Pulliam et al., 2025; van Laarhoven et al., 2018), surface dyslexia co-occurs with anomia in early stage semantic dementia (Binney et al., 2016), in developmental anomia (Gvion and Friedmann, 2016), and in acquired anomia (Gvion and Friedmann, 2013). Furthermore, unlike stimulation of VWFA, which impairs reading, but not visual naming (Hirshorn et al., 2016; Mani et al., 2008; Woolnough and Tandon, 2024), disruptive stimulation of the rest of BTLS, in anterior and medial VTC, impairs both reading and picture naming (Forseth et al., 2018; Ueno et al., 2018; Woolnough and Tandon, 2024). One hypothesis from this model is that those patients with the greatest visual anomia may also display surface dyslexia.

There is a good deal of nuance when it comes to lexicality and semantics and the assignment of “lexicosemantics” to a single pathway is likely an oversimplification. Semantic knowledge is grounded in the systems involved in processing actions, perceptions, and emotions (Martin, 2016), many, though not all, of which lie along the ventral temporal-to-frontal pathway along the inferior longitudinal fasciculus and inferior frontal occipital fasciculus. It is debated as to whether there is a “amodal semantic hub” in the anterior temporal lobes in addition to this distributed semantic representation as semantic knowledge is impacted in temporal lobe degeneration (Ralph et al., 2017), however others have argued that these deficits are seen primarily only once degeneration impacts much of the temporal lobe beyond the anterior portion (Simmons and Martin, 2009).

Lexicality is also not a singular process and while anterior temporal lobe degeneration causes surface dyslexia and naming errors, studies demonstrate that lesions of regions more consistent with what we have termed the orthographic-to-phonological pathway can also cause surface dyslexia (Binder et al., 2016). There is some evidence that there may be a division between proper and common name representations, with proper names having more ventral and anterior temporal representation and common name representations emphasizing aspects of the posterior middle temporal gyrus (Burkhardt et al., 2025). Exception word reading may lean on both of these types of lexical representations to some degree, which is why surface dyslexia is seen for damage to both (Binder et al., 2016; Wilson et al., 2012, 2009). This is often the case with multi-pathway systems, as the clean divisions in models are not so neat due to the brain’s interactivity. This consideration may be especially relevant in understanding the role of the posterior middle temporal gyrus in reading and picture naming. This region receives connections from the orthographic-to-phonological pathway and the orthographic-to-lexicosemantic pathway. It has been implicated in aspects of lexical-semantic processing associated with both spoken and printed words and demonstrates sensitivity to lexical-phonological factors as well. Damage to this region affects speech comprehension, auditory word-picture matching, and visual word and object naming. These findings suggest that posterior middle temporal gyrus may integrate information across pathways, for instance by capturing information about how lexical-phonological representations tend to statistically co-occur as we use language to communicate about objects. Further studies are required to directly compare the similarity and differences of deficits and processes found for the anterior temporal lobes and the posterior middle temporal gyrus as both have been associated with object naming deficits and surface dyslexia.

One issue is that while much of our knowledge of arise from the deficits that results from resection, stroke, degeneration, or other types of brain damage, plasticity following these insults is complex and variable, and often numerous examples exist of relatively unimpaired language after insults to what seem to be regions critical to these processes (Andrews et al., 2023; Binder, 2017). Complexity arises because these different kinds of damage can implicate different anatomical localizations for deficits (Binder et al., 2016; Domoto-Reilly et al., 2012; Wilson et al., 2012, 2009), potentially because they differ in their clinical course, how much plasticity is possible after different types of insult, and the degree to which damage is primarily restricted to grey versus white matter.

## 9. Stimulation in VTC and reading

Intracranial electrical stimulation studies in individuals undergoing surgical treatment for epilepsy provide the opportunity to causally assess the specificity of VTC regions for reading and test predictions from neural models of reading. As discussed above, stimulation of the VWFA disrupts word reading, but not object or face naming (Hirshorn et al., 2016; Mani et al., 2008; Woolnough and Tandon, 2024); whereas stimulation of medial and anterior aspects of VTC/the BTLS disrupts both (Forseth et al., 2018; Woolnough and Tandon, 2024) (there are also sites in VTC that disrupt object naming, but not word reading (Woolnough and Tandon, 2024)). This dissociation of word reading and face naming disruptions is consistent with the model in Fig. 2, as VWFA acts as a key word selective hub in VTC that feeds both the orthographic-to-phonological and orthographic-to-lexicosemantic pathways. Thus, stimulation of VWFA should disproportionately disrupt word reading, but not object naming, whereas stimulation of other parts of the BTLS along the potentially shared word reading and object naming lexicosemantic pathway disrupts both. Yet to be assessed is the hypothesis that there are aspects of the orthographic-to-phonological pathway that disrupts word reading and lip/speech reading. For example, are there areas where stimulation causes word reading to be disrupted and disrupts the McGurk effect, causing people to perceive the auditory information without interference from the lip forms? Similarly, this hypothesis could be tested by examining whether there are regions where stimulation causes word reading to be disrupted and disrupts people ability to use visual information to help parse speech in noise.

## 10. Reading in the brains of individuals who are blind

**Fig. 5.**
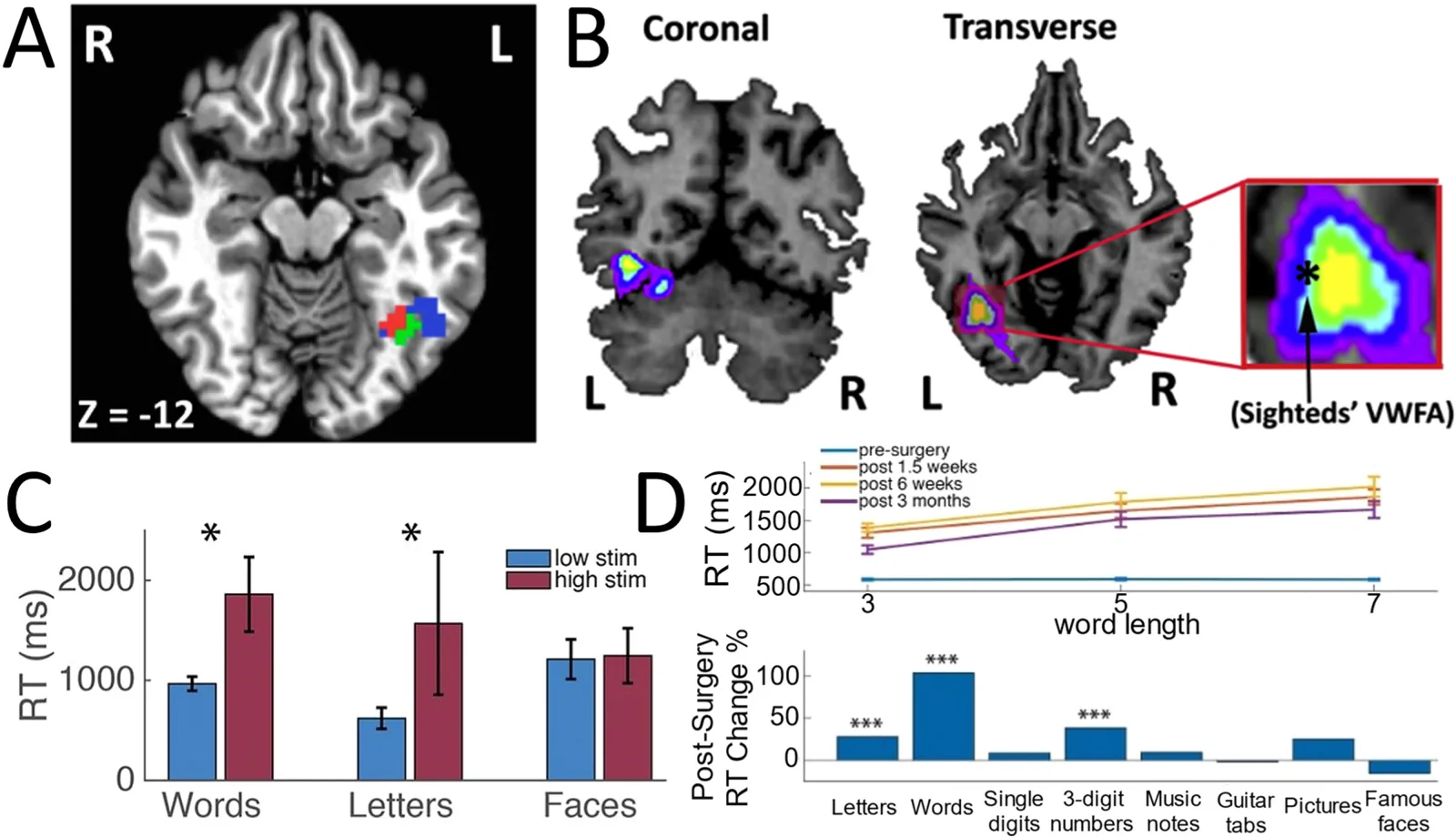
Functional properties of VWFA. **A)** Participants learning to read artificial orthographies composed of houses demonstrated significant changes in neural activation relative to untrained Korean orthography (blue) in areas overlapping (red) with VWFA (green) (adapted from Martin et al. 2019). **B)** Parametric map of braille reading over nonsense braille reading in 8 blind individuals (from Reich et al. 2011). Peak BOLD activations in blind individuals reading braille were highly consistent with peak activations in sighted individuals performing an orthographic task both at the group level and across individuals. **C)** Electrical brain stimulation through electrode contacts implanted in VWFA profoundly delayed word and letter naming but not famous face naming (adapted from Hirshorn et al. 2016). **D)** Focal resection including VWFA caused profound and persistent alexia-like impairments in word naming which is exacerbated by word-length (adapted from Hirshorn et al. 2016). Response time impairments were greatest for words, but also extended to letters and numbers but not faces, musical notation, or pictures of other objects.

Tactile reading in individuals who are blind presents an important test for models of reading. Neuroimaging studies have shown that VWFA is activated when reading braille (Reich et al., 2011; Siuda-Krzywicka et al., 2016). There is some debate about the nature of this activation. Most of the debate has focused on understanding why a visual area is active during tactile reading and approaching the question as an issue of neuroplasticity in the context of sensory deprivation and the route to the VWFA is likely different in individuals whose blindness is congenital versus acquired. For instance, one potential explanation is that activation of VWFA in individuals who are blind is a result of direct top-down activation of VWFA (Fig. 5). Top-down activation may be particularly relevant in individuals with acquired blindness as these individuals retain vivid visual imagery and may activate parts of the visual cortex though through the conscious or unconscious visual imagery of letters and words when reading braille. Activation of VWFA may result from “visual” information arising from this visual imagery either directly activating VWFA or traveling down the ventral stream to the VWFA (Hahamy et al., 2021). The extent to which activation to the VWFA is related to access to the broader language system, top-down activation suggests that VWFA’s anatomical connectivity makes it well suited to contribute to printed language processing even without visual input.

Alternatively, VWFA activation in individuals who are blind may occur via bottom-up projections from the ventral stream, as it does in sighted individuals. In congenital blindness, non-visual information may reach early visual cortex directly from subcortical regions due to a lack of visual-activity-dependent pruning (Maurer et al., 2012). It has been well documented that at birth the brain has many more connections than it does in adulthood, with connections pruned away during development in an, at least, partially activity dependent fashion. It has been hypothesized that the cortex is more multimodal at birth compared to adulthood (Dehay et al., 1988; Maurer et al., 2012). In sighted individuals, non-visual connections to visual cortex may be pruned away due to visual activity, whereas in individuals who are congenitally blind, non-visual inputs into these regions could remain due to the lack of visual activity. Consistent with this hypothesis, studies have shown very rapid activation of early visual cortex (Müller et al., 2019) and while there is a statistically significant reduction in thalamus-primary visual cortex white matter, the absolute reduction is moderate (Karlen et al., 2006; Marins et al., 2023; Ptito et al., 2008), leaving pathways for non-visual sensory information to enter primary visual cortex. This would provide a bottom-up ventral stream pathway for braille to activate VWFA in individuals who are congenitally blind and access to the broad language system by braille may progress from the VWFA as in Fig. 2. This hypothesis is also consistent with theory that synesthesia arises due to abnormal neural pruning (Maurer et al., 2020; Ramachandran and Hubbard, 2001). Relatively few studies have compared tactile reading and language in acquired versus congenital blindness, which would be important to determine if non-visual activation of VWFA occur due to different processes in these populations.

Overall, VWFA activation in individuals who are blind is consistent with the idea that this area is involved in reading due to its connectivity with both the language system and VTC pathways (Fig. 2).

### 10.1. Extant models of VWFA

Several extant hypotheses detail VWFA’s location and role in the reading system. Broadly, these hypotheses can be categorized by two factors: 1. whether VWFA processing is specific to orthography or just particularly useful for orthography; 2. whether the location and selectivity of VWFA is driven primarily by bottom-up processing of visual features or by both these bottom-up interactions and top-down interactions with other language areas.

The first of these hypotheses exclusively emphasizes the role of bottom-up processing (Arcaro et al., 2020, 2019; Arcaro and Livingstone, 2021, 2017; Srihasam et al., 2014), suggesting that VWFA is specialized for processing foveal information, and potentially certain shapes, due to its connectivity pattern with earlier visual cortex. These visual features are in turn important for the perception of written words (Vogel et al., 2012). This functional specificity is conferred by “proto--maps” existing at birth, which organize sensory, motor, and association cortices according to low-level features, like topography and tonotopy (Arcaro and Livingstone, 2021).

Challenging this model, neuroimaging in children has demonstrated that the region which eventually becomes VWFA demonstrates increased connectivity to the broader language network prior to learning how to read (Fig. 2A) (Li et al., 2020; Saygin et al., 2016). A model in which only bottom-up connections and protomaps drive selectivity dismisses the relevance of connectivity to the language system that precedes functional specialization of VWFA. Furthermore, selective VWFA responses have also been demonstrated while deaf individuals read sign language or fingerspelling (Waters et al., 2007), after individuals have been trained to associate artificial orthographies composed of objects such as faces and houses with phonology and lexicosemantics (Fig. 5A) (Martin et al., 2019; Moore et al., 2014), and in blind individuals reading braille (Fig. 5B) (Reich et al., 2011; Siuda-Krzywicka et al., 2016). These studies argue for a remarkable degree of local plasticity in the types of orthographic features VWFA can process. The concept of proto-maps fails to parsimonously explain these results, since it fails to explicate the low-level visual features that unify these diverse stimuli and the correspondences between retinotopy, tonotopy, and somatotopy that link braille, sign language, spoken language, and reading. A model in which top-down and bottom-up circuitry evolved for social communication and object naming is easier to reconcile with these data.

Another characterization of VWFA as a non-orthographic-specific center, highlights the relationship between visual word and face processing networks bilaterally (Behrmann and Plaut, 2020, 2013). Face- and word-selective regions in VTC are remarkably close together although face and word stimuli differ in their visual appearance, their relevance to our evolutionary ancestors, and the higher-order brain networks that process them (Op de Beeck et al., 2019). This model argues that this is because both systems require input from regions with foveal receptive fields and involve fine-scale visual discrimination (Blauch et al., 2022). In these models, during the acquisition of literacy, VWFA neurons edge-out face-selective representations in the left hemisphere causing a hemispheric bias due to proximity constraints with other left-lateralized language areas (Plaut and Behrmann, 2011). After the acquisition of literacy, both hemispheres contribute to face and word processing in this model, though not equally (Behrmann and Plaut, 2014).

However, face-selective VTC in preliterate children has significantly less structural connectivity to perisylvian language regions compared to the parts of VTC that become word-selective upon the acquisition of literacy (Li et al., 2020; Saygin et al., 2016) (Fig. 2A). Additionally, word-selective responses recorded intracranially from literate adults demonstrate that some left word-selective regions show no degree of face selectivity (Boring et al., 2021; Hagen et al., 2021) and cortical stimulation and lesioning of VWFA selectively impairs reading but not famous face recognition (Fig. 5C & D) (Hirshorn et al., 2016; Sabsevitz et al., 2020). Furthermore, the timing of word-selective response in the left and right hemisphere are very different, suggesting qualitative, rather than graded, differences in their contributions to reading (Fig. 4A) (Boring et al., 2021). Right hemisphere word-selective responses may be involved in integrating words into context, reflected in the N400 event-related potential component recorded using non-invasive EEG (Federmeier and Kutas, 1999), but not necessarily the bottom-up processing of word forms (White et al., 2019). While these results speak against joint circuits for face and word processing based on shared visual processing demands (Behrmann and Plaut, 2020, 2014), the proximity and analogous visuolinguistic transformations required to process words and communicative linguistic information from the face (rather than analogous visual computations) may help explain the function and localization of reading pathways.

The “interactive account” of VWFA suggests that it contains “general purpose analyzers of visual forms” (Price and Devlin, 2011) used for object naming more broadly, not orthography per se. These non-specific, bottom-up feature detectors derive their observed selectivity for words through dynamic interactions with brain regions that contain phonological and semantic information established during the acquisition of language (Price and Devlin, 2011, 2003). Thus the theory explains the consistent localization of VWFA across individuals due to its evolutionarily preserved role in visual naming (Price and Devlin, 2011), and why prereading children already demonstrate preferential connectivity between VWFA and the broader language network (Li et al., 2020; Saygin et al., 2016). However, disrupting VWFA activity through lesions or cortical stimulation can lead to selective deficits in reading but not object naming (Fig. 5C & D) (Gaillard et al., 2006; Hirshorn et al., 2016; Mani et al., 2008; Sabsevitz et al., 2020; Woolnough and Tandon, 2024). Further, intracranial and fMRI studies of VWFA demonstrate preferential responses to words compared to other nameable objects (Centanni et al., 2017; Hirshorn et al., 2016; Szwed et al., 2011), making it implausible that VWFA underlies all object naming.

Finally, the “cultural recycling” hypothesis also argues for the importance of bottom-up and top-down connectivity in defining VWFA’s localization but stresses that its role is specific to orthography (Dehaene and Cohen, 2007; Hannagan et al., 2015). In this model, the VWFA occupies a similar region across people due to that region’s position in a circuit whose original function is sufficiently close to that required by reading (like social communication), but that original function is recycled in service of reading. Early versions of the cultural recycling hypothesis emphasized compatibility of local cortical computations (Dehaene and Cohen, 2007), though an updated version of this hypothesis incorporated recycling of long-range connections as well (Hannagan et al., 2015). One open question in these models is where the original functions of the VWFA go after we learn to read, as these functions are not lost after the acquisition of literacy.

The model of VWFA presented here (Fig. 2C) suggests an adaptation of preexisting circuits for naming, semantics, and phonology and stresses the importance of both top-down and bottom-up interactions in defining VWFA selectivity. This model is broadly consistent with the cultural recycling hypothesis. It is notable that a dissociation between word reading and lip/speech reading and gesture perception has not been examined in VWFA. The cultural recycling hypothesis takes a strong stand that the recycling of neural function through learning during development displaces the other functions of a region (Dehaene and Cohen, 2007). The model in Fig. 2C would not require any potential lip/speech reading or gesture perception functions of the VWFA to be displaced, and is thus would also be consistent with the redeployment hypothesis (Anderson, 2010).

Furthermore, although the cultural recycling hypothesis acknowledges a role for connectivity, it emphasizes computational compatibility of the region of the brain that is recycled (Dehaene and Cohen, 2007; Hannagan et al., 2015). Conversely, the model here emphasizes the role of connectivity and the compatibility of the types of transformations between representations needed for reading, while placing reduced emphasis on local computational compatibility, in constraining the localization of VWFA. That said, local computational compatibility, driven by inherent neurophysiological properties and the resulting processing capabilities, likely also plays some role in VWFA localization (Grill-Spector and Weiner, 2014). This area of VTC is involved in holistic and configural processing (Bukach et al., 2006; Hirshorn et al., 2016). This is not only true for words, but also for faces in neighboring face-selective regions (Maurer et al., 2002), and other neighboring regions, which develop holistic and configural processing characteristics for stimuli of acquired visual expertise (Bukach et al., 2006). However, local computational compatibility alone lacks explanatory power for why braille reading in blind individuals, sign language in deaf individuals, or faces and houses in individuals trained to read artificial orthographies preferentially activate VWFA (Reich et al., 2011; Waters et al., 2007); rather these findings suggest there is a great deal of plasticity in local computation. Furthermore, recent studies have called into question whether VTC is involved in holistic and configural processing (Ayzenberg and Behrmann, 2022; Jagadeesh and Gardner, 2022). However, the findings that damage to the VWFA causes acquired alexia resulting in letter-by-letter reading (Gaillard et al., 2006; Hirshorn et al., 2016) is supportive of holistic/configural processing in VTC, at least for words. Broadly speaking, what type of local computational compatibility would support visual word reading, braille reading in blind individuals, and be consistent with recycling processes involved in object naming and/or lip/speech reading, beyond a general process like configural or holistic processing is unclear. Local computational consistency would also not explain why VWFA ends up in the part of VTC it does. E.g. much of VTC is thought to participate in aspects of holistic processing for different categories of visual stimuli, but VWFA has specific anatomical localization and connectivity compared, for instance, to object, place, or face selective regions of VTC. In our model, the compatibility of representational transformations through bidirectional anatomical connectivity is central to shaping the reading circuit.

## 11. Concluding remarks

Recent findings support a model in which VWFA is a key hub for orthographic processing by serving as a waypoint for lower-level visual centers to leverage visuo-linguistic processing circuitry present prior to learning to read (Fig. 2). Specifically, the VWFA-lateral temporal and frontal cortex pathway underlying orthophonological mappings for reading leverages existing circuitry for visuo-linguistic mapping between visual representation of mouth forms to phonology (Hannagan et al., 2015). Additionally, the VWFA-BTLS, lateral temporal, and frontal cortex pathway underlying lexicosemantic mappings leverages existing circuitry for visual naming.

The connectivity in this model is fully bidirectional. This is supported both by neuroanatomy, where studies have shown few cortical connections are unidirectional (Kravitz et al., 2013), and by functional studies suggesting recurrence. Specifically, the finding that representations in VWFA shift from being coarse to individuated over the period of hundreds of milliseconds (Boring et al., 2018; Hirshorn et al., 2016) suggests that the mutual constraints applied by orthography, phonology, and lexicosemantics filter through the entire reading system over time. Bidirectional connectivity also provides a pathway for top-down constraints to be applied to the reading process, for example by sentence context (Kutas and Federmeier, 2011), and provides a pathway to explain how braille reading activates VWFA in the blind without visual input (Reich et al., 2011).

More generally, reading may represent a special case of a broad principle for how acquiring expert skills leverages existing brain circuitry. Specifically, the hypothesis presented here suggests that acquiring expertise involves adapting existing neural processing pathways to perform closely related transformations (Veit et al., 2021), whereas representations within brain regions have a remarkable degree of plasticity during skill acquisition (Martin et al., 2019; Moore et al., 2014; Riesenhuber and Glezer, 2017). For example, VWFA can adapt its representations to process artificial orthographies, braille, and sign language (Martin et al., 2019; Moore et al., 2014; Reich et al., 2011; Waters et al., 2007), all of which require similar phonological and lexicosemantic transformations, but very different perceptual representations. The model presented here bears resemblance to theories of cortical specialization that emphasize the role of connectivity between areas (Hannagan et al., 2015; Mahon and Caramazza, 2011; Martin, 2006), though these theories have not necessarily specified critical transformations that organize acquired skills. This model suggests a “compatibility of transformations” hypothesis for organizing the representation of expert skills in the brain, demonstrating a potential principle for how nurture creates neural changes that leverage the intrinsic nature of brain circuits.

## Supplementary Material

1

## 12. Outstanding questions

- How are phonological and lexicosemantic circuits leveraged during reading acquisition to tune the VWFA for orthography?
- Does VWFA contribute to lip/speech reading and/or gesture perception? Do word reading and lip/speech reading and/or gesture perception overlap elsewhere? Does the specific pre-literacy activity or connectivity strength of pathways implicated in lip/speech reading predict the location and/or functional specialization of VWFA once literacy is acquired?
- Are the pathways responsible for lip/speech reading and word reading overlapping after children learn to read or does it subdivide, leaving parallel but non-overlapping pathways for these skills? Similarly, does the pathway for object and face naming subdivide from the lexicosemantic pathway, as is suggested by stimulation of VWFA during famous face and visual object naming?
- What is the role of local computational capacity in shaping the VWFA? How does local neurophysiological and computational processes interact with connectivity to shape the VWFA and the reading circuit as a whole?
- Proper naming and general object naming have been shown to be dissociable in patients with anterior BTLS damage (Abel et al., 2015; Hanley and Kay, 1998). Does exception word naming in BTLS more closely resemble proper naming or general object naming?
- To what degree are the lexicosemantic and orthophonological pathways responsible for object-naming and lip/speech reading innate or shaped by learning?
- A small number of individuals appear to be able to read without hearing words in their minds, and those who are congenitally deaf can achieve expertise in reading. How do the reading pathways in the brain differ in those who do and do not hear words in their minds when reading?
- What is the role of posterior VWFA connectivity via the vertical occipital fasciculus to dorsal visual processing regions, which have been associated with attentional deployment during reading (Chen et al., 2019; H. Komatsu and Wurtz, 1988)? Do these connections, for example, help guide eye movements and attention during the sequential decoding of printed words (especially in early readers), and sentences? What are the neural correlates of different types of reading eye movements, such as regressive saccades?
- What is the balance between bottom-up and top-down connectivity in determining the location of VWFA (i.e., does anatomical connectivity to the visual system or anatomical connectivity to the language system provide more or less constraint)? Furthermore, does local cortical physiology make the VWFA more adept at processing visual word forms than neighboring face- and limb-selective cortex?
- This review suggests a model for how single words are processed in the brain. How are these and extended language networks leveraged to understand phrases, sentences, paragraphs, and narratives during natural reading?

## Appendix A. Supporting information

Supplementary data associated with this article can be found in the online version at doi:10.1016/j.pneurobio.2026.102894.

## Glossary

Alexia: An inability to recognize printed words, usually caused by damage to VTC. Patients with alexia often resort to a letter-by-letter reading strategy, resulting in large response times for reading individual words compared to literate controls
Lip/Speech reading: Colloquially called “lip reading,” more precisely named speech reading in the speech and language literature, this is the perception of mouth forms used to support speech and language perception. Lip/speech reading is particularly helpful for understanding speech in noisy environments and mismatches between mouth forms and what is heard can cause misperceptions, such as in the McGurk Effect
McGurk Effect: A perceptual illusion where speech sounds are misperceived due to a mismatch between auditory speech and visual mouth form cues. This illusion demonstrates how visual perception of mouth forms influences auditory perception of speech
Orthography: system for conveying words in printed form. This includes the formation of words by letters, characters, pictographs, or braille cells
Phonological dyslexia: An acquired reading disorder in which individuals have particular difficulty reading unfamiliar but pronounceable pseudowords, in comparison to their ability to read words they have experienced
Prosopagnosia: An inability to visually recognize faces. Can be the result of VTC damage or congenital abnormality
Semantic dementia: A degenerative neurological disorder primarily affecting the ability of patients to retrieve the meanings of words. The disorder is often accompanied by surface dyslexia and trouble naming objects. It is associated with anterior temporal and/or frontal lobe degeneration and is sometimes referred to as semantic variant primary progressive aphasia
Surface dyslexia: A neurological disorder primarily characterized by regularizing the pronunciation of exception words like yacht, colonel, or island. It is sometimes associated with degeneration or damage to the anterior temporal or frontal lobes. Surface dyslexia is also a common early symptom of semantic dementia
Ventral temporal cortex (VTC): An anatomical region of the human brain consisting of the collateral sulcus, fusiform (occipitotemporal) gyrus, parahippocampal gyrus and anterior temporal lobes. This area contains high-level visual centers important for the visual perception, recognition, and naming of objects

## References

[R1] Abel TJ, Rhone AE, Nourski KV, Kawasaki H, Oya H, Griffiths TD, Howard MA, Tranel D, 2015. Direct physiologic evidence of a heteromodal convergence region for proper naming in human left anterior temporal lobe. J. Neurosci. 35, 1513–1520. 10.1523/JNEUROSCI.3387-14.2015. 25632128 PMC4308598

[R2] Albonico A, Barton JJS, 2017. Face perception in pure alexia: Complementary contributions of the left fusiform gyrus to facial identity and facial speech processing. Cortex 96, 59–72. 10.1016/J.CORTEX.2017.08.029. 28964939

[R3] Alvarez TA, Fiez JA, 2018. Current perspectives on the cerebellum and reading development. Neurosci. Biobehav. Rev. 92, 55–66. 10.1016/j.neubiorev.2018.05.006. 29730484 PMC6078792

[R4] Anderson ML, 2010. Neural reuse: A fundamental organizational principle of the brain. Behav. Brain Sci. 33, 245–266. 10.1017/S0140525X10000853. 20964882

[R5] Andrews JP, Cahn N, Speidel BA, Chung JE, Levy DF, Wilson SM, Berger MS, Chang EF, 2023. Dissociation of Broca’s area from Broca’s aphasia in patients undergoing neurosurgical resections. J. Neurosurg. 138, 847–857. 10.3171/2022.6.JNS2297. 35932264 PMC9899289

[R6] Araki K, Terada K, Usui K, Usui N, Araki Y, Baba K, Matsuda K, Tottori T, Inoue Y, 2015. Bidirectional neural connectivity between basal temporal and posterior language areas in humans. Clin. Neurophysiol. 126, 682–688. 10.1016/J.CLINPH.2014.07.020. 25190148

[R7] Arcaro MJ, Livingstone MS, 2017. A hierarchical, retinotopic proto-organization of the primate visual system at birth. eLife 6, e26196. 10.7554/eLife.26196. 28671063 PMC5495573

[R8] Arcaro MJ, Livingstone MS, 2021. On the relationship between maps and domains in inferotemporal cortex. Nat. Rev. Neurosci. 22, 573–583. 10.1038/s41583-021-00490-4. 34345018 PMC8865285

[R9] Arcaro MJ, Mautz T, Berezovskii VK, Livingstone MS, 2020. Anatomical correlates of face patches in macaque inferotemporal cortex. Proc. Natl. Acad. Sci. USA 117, 32667–32678. 10.1073/PNAS.2018780117/-/DCSUPPLEMENTAL. 33277435 PMC7768718

[R10] Arcaro MJ, Schade PF, Livingstone MS, 2019. Universal Mechanisms and the Development of the Face Network: What You See Is What You Get. Annu. Rev. Vis. Sci. 5, 341–372. 10.1146/annurev-vision-091718-014917. 31226011 PMC7568401

[R11] Aron O, Krieg J, Brissart H, Abdallah C, Colnat-Coulbois S, Jonas J, Maillard L, 2022. Naming impairments evoked by focal cortical electrical stimulation in the ventral temporal cortex correlate with increased functional connectivity. Neurophysiol. Clin. 52, 312–322. 10.1016/J.NEUCLI.2022.06.002. 35777988

[R12] Ayzenberg V, Behrmann M, 2022. Does the brain’s ventral visual pathway compute object shape? Trends Cogn. Sci. 26, 1119–1132. 10.1016/j.tics.2022.09.019. 36272937 PMC11669366

[R13] Bédos Ulvin L, Jonas J, Brissart H, Colnat-Coulbois S, Thiriaux A, Vignal JP, Maillard L, 2017. Intracerebral stimulation of left and right ventral temporal cortex during object naming. Brain Lang. 175, 71–76. 10.1016/J.BANDL.2017.09.003. 29024845

[R14] Behrmann M, Plaut DC, 2013. Distributed circuits, not circumscribed centers, mediate visual recognition. Trends Cogn. Sci. 17, 210–219. 10.1016/j.tics.2013.03.007. 23608364

[R15] Behrmann M, Plaut DC, 2014. Bilateral hemispheric processing of words and faces: evidence from word impairments in prosopagnosia and face impairments in pure alexia. Cereb. Cortex 24, 1102–1118. 10.1093/cercor/bhs390. 23250954

[R16] Behrmann M, Plaut DC, 2020. Hemispheric organization for visual object recognition: a theoretical account and empirical evidence. Perception 49, 373–404. 10.1177/0301006619899049. 31980013 PMC9944149

[R17] Binder JR, 2017. Current Controversies on Wernicke’s Area and its Role in Language. Curr. Neurol. Neurosci. Rep. 17, 58. 10.1007/s11910-017-0764-8. 28656532

[R18] Binder JR, Pillay SB, Humphries CJ, Gross WL, Graves WW, Book DS, 2016. Surface errors without semantic impairment in acquired dyslexia: a voxel-based lesion–symptom mapping study. Brain 139, 1517–1526. 10.1093/brain/aww029. 26966139 PMC5006249

[R19] Binney RJ, Henry ML, Babiak M, Pressman PS, Santos-Santos MA, Narvid J, Mandelli ML, Strain PJ, Miller BL, Rankin KP, Rosen HJ, Gorno-Tempini ML, 2016. Reading words and other people: A comparison of exception word, familiar face and affect processing in the left and right temporal variants of primary progressive aphasia. Cortex 82, 147–163. 10.1016/j.cortex.2016.05.014. 27389800 PMC4969161

[R20] Binney RJ, Parker GJM, Lambon Ralph MA, 2012. Convergent connectivity and graded specialization in the rostral human temporal lobe as revealed by diffusion-weighted imaging probabilistic tractography. J. Cogn. Neurosci. 24, 1998–2014. 10.1162/JOCN_A_00263. 22721379

[R21] Blauch NM, Behrmann M, Plaut DC, 2022. A connectivity-constrained computational account of topographic organization in primate high-level visual cortex. Proc. Natl. Acad. Sci. USA 119, e2112566119. 10.1073/pnas.2112566119. 35027449 PMC8784138

[R22] Boring MJ, Hirshorn EA, Li Y, Ward MJ, Richardson RM, Fiez JA, Ghuman AS, 2018. Left mid-ventral temporal cortex interacts with early visual cortex and the anterior temporal lobe to support word individuation. bioRxiv, 411579. 10.1101/411579.

[R23] Boring MJ, Silson EH, Ward MJ, Richardson RM, Fiez JA, Baker CI, Ghuman AS, 2021. Multiple adjoining word- and face-selective regions in ventral temporal cortex exhibit distinct dynamics. J. Neurosci. 41, 6314–6327. 10.1523/JNEUROSCI.3234-20.2021. 34099511 PMC8287994

[R24] Bouhali F, Schotten M.T. de, Pinel P, Poupon C, Mangin J-F, Dehaene S, Cohen L, 2014. Anatomical Connections of the Visual Word Form Area. J. Neurosci. 34, 15402–15414. 10.1523/JNEUROSCI.4918-13.2014. 25392507 PMC6608451

[R25] Brédart S, 2017. The cognitive psychology and neuroscience of naming people. Neurosci. Biobehav. Rev. 83, 145–154. 10.1016/J.NEUBIOREV.2017.10.008. 29038031

[R26] Brownsett SLE, Wise RJS, 2010. The Contribution of the Parietal Lobes to Speaking and Writing. Cereb. Cortex 20, 517–523. 10.1093/cercor/bhp120. 19531538 PMC2820696

[R27] Bub DN, Arguin M, Lecours AR, 1993. Jules Dejerine and His Interpretation of Pure Alexia. Brain Lang. 45, 531–559. 10.1006/BRLN.1993.1059. 8118672

[R28] Bukach CM, Gauthier I, Tarr MJ, 2006. Beyond faces and modularity: the power of an expertise framework. Trends Cogn. Sci. 10, 159–166. 10.1016/J.TICS.2006.02.004. 16516534

[R29] Burkhardt E, Duffau H, Herbet G, 2025. Networks for proper versus common name retrieval: insights from causal mapping and new hypotheses. Brain Commun. 7, fcaf256. 10.1093/braincomms/fcaf256. 40661329 PMC12256651

[R30] Calvert GA, Bullmore ET, Brammer MJ, Campbell R, Williams SCR, McGuire PK, Woodruff PWR, Iversen SD, David AS, 1997. Activation of auditory cortex during silent lipreading. Science 276, 593–596. 10.1126/science.276.5312.593. 9110978

[R31] Campbell R, Landis T, Regard M, 1986. Face recognition and lipreading: A neurological dissociation. Brain 109, 509–521. 10.1093/brain/109.3.509. 3719288

[R32] Castles A, Coltheart M, 1993. Varieties of developmental dyslexia. Cognition 47, 149–180. 10.1016/0010-0277(93)90003-E. 8324999

[R33] Centanni TM, King LW, Eddy MD, Whitfield-Gabrieli S, Gabrieli JDE, 2017. Development of sensitivity versus specificity for print in the visual word form area. Brain Lang. 170, 62–70. 10.1016/J.BANDL.2017.03.009. 28411527

[R34] Chen L, Wassermann D, Abrams DA, Kochalka J, Gallardo-Diez G, Menon V, 2019. The visual word form area (VWFA) is part of both language and attention circuitry. Nat. Commun. 10. 10.1038/S41467-019-13634-Z. PMC6898452 31811149

[R35] Cloppenborg T, Mertens M, Hopf JL, Kalbhenn T, Bien CG, Woermann FG, Polster T, 2021. Reading and the visual word form area (VWFA) – Management and clinical experience at one epilepsy surgery center. Epilepsy Behav. 124, 108274. 10.1016/j.yebeh.2021.108274. 34536734

[R36] Cohen L, Dehaene S, Naccache L, Lehéricy S, Dehaene-Lambertz G, Hénaff MA, Michel F, 2000. The visual word form area. Spat. Tempo Charact. Initial Stage Read. Norm. Subj. Posterior SplitBrain Patients Brain 123, 291–307. 10.1093/brain/123.2.291. 10648437

[R37] Coltheart M, Rastle K, Perry C, Langdon R, Ziegler J, 2001. DRC: A dual route cascaded model of visual word recognition and reading aloud. Psychol. Rev. 108, 204–256. 10.1037/0033-295X.108.1.204. 11212628

[R38] Damasio AR, 1979. Wernicke’s Works on Aphasia: A Sourcebook and Review, 324 Arch. Neurol. 36, 324. 10.1001/archneur.1979.00500410102028.

[R39] De Gelder B, Vroomen J, 1998. Impaired speech perception in poor readers: Evidence from hearing and speech reading. Brain Lang. 64, 269–281. 10.1006/brln.1998.1973. 9743542

[R40] Dehaene S, Cohen L, 2007. Cultural Recycling of Cortical Maps. Neuron 56, 384–398. 10.1016/J.NEURON.2007.10.004. 17964253

[R41] Dehaene SC, Le Clec’H G, Poline J-B, Le Bihan D, Cohen L, 2002. The visual word form area: a prelexical representation of visual words in the fusiform gyrus. Neuroreport 13, 321–325. 11930131 10.1097/00001756-200203040-00015

[R42] Dehay C, Kennedy H, Bullier J, 1988. Characterization of transient cortical projections from auditory, somatosensory, and motor cortices to visual areas 17, 18, and 19 in the kitten. J. Comp. Neurol. 272, 68–89. 10.1002/CNE.902720106. 2454978

[R43] Devlin JT, Russell RP, Davis MH, Price CJ, Wilson J, Moss HE, Matthews PM, Tyler LK, 2000. Susceptibility-Induced Loss of Signal: Comparing PET and fMRI on a Semantic Task. NeuroImage 11, 589–600. 10.1006/NIMG.2000.0595. 10860788

[R44] Domoto-Reilly K, Sapolsky D, Brickhouse M, Dickerson BC, 2012. Naming impairment in Alzheimer’s disease is associated with left anterior temporal lobe atrophy. NeuroImage 63, 348–355. 10.1016/j.neuroimage.2012.06.018. 22728617 PMC3665400

[R45] Erickson LC, Zielinski BA, Zielinski JEV, Liu G, Turkeltaub PE, Leaver AM, Rauschecker JP, 2014. Distinct cortical locations for integration of audiovisual speech and the McGurk effect. Front. Psychol. 5, 534. 10.3389/FPSYG.2014.00534. 24917840 PMC4040936

[R46] Federmeier KD, Kutas M, 1999. Right words and left words: Electrophysiological evidence for hemispheric differences in meaning processing. Cogn. Brain Res. 8, 373–392. 10.1016/S0926-6410(99)00036-1. 10556614

[R47] Forseth KJ, Kadipasaoglu CM, Conner CR, Hickok G, Knight RT, Tandon N, 2018. A lexical semantic hub for heteromodal naming in middle fusiform gyrus. Brain 141, 2112–2126. 10.1093/brain/awy120. 29860298 PMC6365955

[R48] Francisco AA, Takashima A, McQueen JM, van den Bunt M, Jesse A, Groen MA, 2018. Adult dyslexic readers benefit less from visual input during audiovisual speech processing: fMRI evidence. Neuropsychologia 117, 454–471. 10.1016/j.neuropsychologia.2018.07.009. 29990508

[R49] Friederici AD, 2011. The Brain Basis of Language Processing: From Structure to Function. Physiol. Rev. 91, 1357–1392. 10.1152/physrev.00006.2011. 22013214

[R50] Gaillard R, Naccache L, Pinel P, Clémenceau S, Volle E, Hasboun D, Dupont S, Baulac M, Dehaene S, Adam C, Cohen L, 2006. Direct intracranial, fMRI, and lesion evidence for the causal role of left inferotemporal cortex in reading. Neuron 50, 191–204. 10.1016/j.neuron.2006.03.031. 16630832

[R51] Ghuman AS, Bar M, Dobbins IG, Schnyer DM, 2008. The effects of priming on frontal-temporal communication. Proc. Natl. Acad. Sci. USA 105, 8405–8409. 10.1073/pnas.0710674105. 18541919 PMC2448849

[R52] Ghuman AS, Fiez JA, 2018. Parcellating the structure and function of the reading circuit. Proc. Natl. Acad. Sci. USA 115, 10542–10544. 10.1073/pnas.1814648115. 30275333 PMC6196548

[R53] Ghuman AS, Martin A, 2019. Dynamic Neural Representations: An Inferential Challenge for fMRI. Trends Cogn. Sci. 23, 534–536. 10.1016/j.tics.2019.04.004. 31103440 PMC6691971

[R54] Giampiccolo D, Duffau H, 2022. Controversy over the temporal cortical terminations of the left arcuate fasciculus: a reappraisal. Brain 145, 1242–1256. 10.1093/brain/awac057. 35142842

[R55] Glezer LS, Jiang X, Riesenhuber M, 2009. Evidence for Highly Selective Neuronal Tuning to Whole Words in the “Visual Word Form Area.”. Neuron 62, 199–204. 10.1016/j.neuron.2009.03.017. 19409265 PMC2706007

[R56] Graves WW, Grabowski TJ, Mehta S, Gordon JK, 2007. A Neural Signature of Phonological Access: Distinguishing the Effects of Word Frequency from Familiarity and Length in Overt Picture Naming. J. Cogn. Neurosci. 19, 617–631. 10.1162/jocn.2007.19.4.617. 17381253

[R57] Grill-Spector K, Weiner KS, 2014. The functional architecture of the ventral temporal cortex and its role in categorization. Nat. Rev. Neurosci. 15, 536–548. 10.1038/nrn3747. 24962370 PMC4143420

[R58] Groen IIA, Dekker TM, Knapen T, Silson EH, 2022. Visuospatial coding as ubiquitous scaffolding for human cognition. Trends Cogn. Sci. 26, 81–96. 10.1016/j.tics.2021.10.011. 34799253

[R59] Grotheer M, Yeatman J, Grill-Spector K, 2021. White matter fascicles and cortical microstructure predict reading-related responses in human ventral temporal cortex. NeuroImage 227, 117669. 10.1016/j.neuroimage.2020.117669. 33359351 PMC8416179

[R60] Gullick MM, Booth JR, 2015. The direct segment of the arcuate fasciculus is predictive of longitudinal reading change. Dev. Cogn. Neurosci. 13, 68–74. 10.1016/J.DCN.2015.05.002. 26011750 PMC4480913

[R61] Gvion A, Friedmann N, 2013. Developmental and Acquired Surface Dyslexia and Anomia as a Result of a Shared Deficit in Phonological Output Lexicon. Procedia Soc. Behav. Sci. 94, 205–206. 10.1016/j.sbspro.2013.09.101.

[R62] Gvion A, Friedmann N, 2016. A principled relation between reading and naming in acquired and developmental anomia: Surface dyslexia following impairment in the phonological output lexicon. Front. Psychol. 7, 340. 10.3389/fpsyg.2016.00340. 27065897 PMC4811952

[R63] Hagen S, Lochy A, Jacques C, Maillard L, Colnat-Coulbois S, Jonas J, Rossion B, 2021. Dissociated face- and word-selective intracerebral responses in the human ventral occipito-temporal cortex. Brain Struct. Funct. 226, 3031–3049. 10.1007/S00429-021-02350-4. 34370091 PMC8541991

[R64] Hahamy A, Wilf M, Rosin B, Behrmann M, Malach R, 2021. How do the blind ‘see’? The role of spontaneous brain activity in self-generated perception. Brain 144, 340–353. 10.1093/BRAIN/AWAA384. 33367630 PMC7880672

[R65] Hanley JR, Kay J, 1998. Proper name anomia for the names of people: Functionally dissociable impairments? Cortex 34, 155–158. 10.1016/S0010-9452(08)70745-7. 9534002

[R66] Hannagan T, Amedi A, Cohen L, Dehaene-Lambertz G, Dehaene S, 2015. Origins of the specialization for letters and numbers in ventral occipitotemporal cortex. Trends Cogn. Sci. 19, 374–382. 10.1016/J.TICS.2015.05.006. 26072689

[R67] Hasson U, Levy I, Behrmann M, Hendler T, Malach R, 2002. Eccentricity Bias as an Organizing Principle for Human High-Order Object Areas. Neuron 34, 479–490. 10.1016/S0896-6273(02)00662-1. 11988177

[R68] Hauswald A, Lithari C, Collignon O, Leonardelli E, Weisz N, 2018. A Visual Cortical Network for Deriving Phonological Information from Intelligible Lip Movements. Curr. Biol. 28, 1453–1459.e3. 10.1016/J.CUB.2018.03.044. 29681475 PMC5956463

[R69] Hermann BP, Chelune GJ, Loring DW, Trenerry MR, Perrine K, Barr W, Strauss E, Westerveld M, 1999. Visual confrontation naming following left anterior temporal lobectomy: A comparison of surgical approaches. Neuropsychology 13, 3–9. 10.1037/0894-4105.13.1.3. 10067770

[R70] Hickok G, Poeppel D, 2007. The cortical organization of speech processing. Nat. Rev. Neurosci. 8, 393–402. 10.1038/nrn2113. 17431404

[R71] Hirshorn EA, Li Y, Ward MJ, Richardson RM, Fiez JA, Ghuman AS, 2016. Decoding and disrupting left midfusiform gyrus activity during word reading. Proc. Natl. Acad. Sci. USA 113, 8162–8167. 10.1073/pnas.1604126113. 27325763 PMC4961146

[R72] Hodges JR, Patterson K, Oxbury S, Funnell E, 1992. Semantic dementia: Progressive fluent aphasia with temporal lobe atrophy. Brain 115, 1783–1806. 10.1093/brain/115.6.1783. 1486461

[R73] Hoffman P, Lambon Ralph MA, Woollams AM, 2015. Triangulation of the neurocomputational architecture underpinning reading aloud. Proc. Natl. Acad. Sci. USA 112, E3719–E3728. 10.1073/pnas.1502032112. 26124121 PMC4507229

[R74] Jacques C, Witthoft N, Weiner KS, Foster BL, Rangarajan V, Hermes D, Miller KJ, Parvizi J, Grill-Spector K, 2016. Corresponding ECoG and fMRI category-selective signals in human ventral temporal cortex. Neuropsychologia 83, 14–28. 10.1016/j.neuropsychologia.2015.07.024. 26212070 PMC4724347

[R75] Jagadeesh AV, Gardner JL, 2022. Texture-like representation of objects in human visual cortex. Proc. Natl. Acad. Sci. U. S. A. 119, e2115302119. 10.1073/pnas.2115302119. 35439063 PMC9169962

[R76] Kaestner E, Stasenko A, Ben-Haim S, Shih J, Paul BM, McDonald CR, 2022. The importance of basal-temporal white matter to pre- and post-surgical naming ability in temporal lobe epilepsy. NeuroImage Clin. 34, 102963. 10.1016/j.nicl.2022.102963. 35220106 PMC8888987

[R77] Karlen SJ, Kahn DM, Krubitzer L, 2006. Early blindness results in abnormal corticocortical and thalamocortical connections. Neuroscience 142, 843–858. 10.1016/j.neuroscience.2006.06.055. 16934941

[R78] Komatsu Hidehiko, Wurtz RH, 1988. Relation of cortical areas MT and MST to pursuit eye movements. I. Localization and visual properties of neurons. J. Neurophysiol. 60, 580–603. 3171643 10.1152/jn.1988.60.2.580

[R79] Komatsu H, Wurtz RH, 1988. Relation of cortical areas MT and MST to pursuit eye movements. I. Localization and visual properties of neurons. J. Neurophysiol. 60, 580–603. 10.1152/JN.1988.60.2.580. 3171643

[R80] Koubeissi MZ, Lesser RP, Sinai A, Gaillard WD, Franaszczuk PJ, Crone NE, 2012. Connectivity between perisylvian and bilateral basal temporal cortices. Cereb. Cortex 22, 918–925. 10.1093/cercor/bhr163. 21715651 PMC3306577

[R81] Kravitz DJ, Saleem KS, Baker CI, Ungerleider LG, Mishkin M, 2013. The ventral visual pathway: an expanded neural framework for the processing of object quality. Trends Cogn. Sci. 17, 26–49. 10.1016/j.tics.2012.10.011. 23265839 PMC3532569

[R82] Kutas M, Federmeier KD, 2011. Thirty years and counting: Finding meaning in the N400 component of the event related brain potential (ERP). Annu. Rev. Psychol. 62, 621–647. 10.1146/ANNUREV.PSYCH.093008.131123. 20809790 PMC4052444

[R83] Lerma-Usabiaga G, Carreiras M, Paz-Alonso PM, 2018. Converging evidence for functional and structural segregation within the left ventral occipitotemporal cortex in reading. Proc. Natl. Acad. Sci. USA 115, E9981–E9990. 10.1073/pnas.1803003115. 30224475 PMC6196482

[R84] Li J, Osher DE, Hansen HA, Saygin ZM, 2020. Innate connectivity patterns drive the development of the visual word form area. Sci. Rep. 2020 10 (1 10), 1–12. 10.1038/s41598-020-75015-7. PMC7582172 33093478

[R85] Liuzzi AG, Bruffaerts R, Vandenberghe R, 2019. The medial temporal written word processing system. Cortex 119, 287–300. 10.1016/j.cortex.2019.05.002. 31174078

[R86] Luders HO, Lesser RP, Hahn J, Dinner DS, Morris HH, Resor S, Harrison M, 1991. Basal temporal language area. Brain 114, 743–754. 2043946 10.1093/brain/114.2.743

[R87] Lüders H, Lesser RP, Hahn J, Dinner DS, Morris HH, Wyllie E, Godoy J, 1991. Basal temporal language area. Brain 114, 743–754. 10.1093/brain/114.2.743. 2043946

[R88] Mahon BZ, Caramazza A, 2011. What drives the organization of object knowledge in the brain? Trends Cogn. Sci. 15, 97–103. 10.1016/j.tics.2011.01.004. 21317022 PMC3056283

[R89] Mani J, Diehl B, Piao Z, Schuele SS, Lapresto E, Liu P, Nair DR, Dinner DS, Lüders HO, 2008. Evidence for a basal temporal visual language center: Cortical stimulation producing pure alexia. Neurology 71, 1621–1627. 10.1212/01.wnl.0000334755.32850.f0. 19001252

[R90] Marins TF, Russo M, Rodrigues EC, Monteiro M, Moll J, Felix D, Bouzas J, Arcanjo H, Vargas CD, Tovar-Moll F, 2023. Reorganization of thalamocortical connections in congenitally blind humans. Hum. Brain Mapp. 44, 2039–2049. 10.1002/hbm.26192. 36661404 PMC9980890

[R91] Marstaller L, Burianová H, 2014. The multisensory perception of co-speech gestures – A review and meta-analysis of neuroimaging studies. J. Neurolinguist. 30, 69–77. 10.1016/J.JNEUROLING.2014.04.003.

[R92] Martin A, 2006. Shades of Déjerine—Forging a Causal Link between the Visual Word Form Area and Reading. Neuron 50, 173–175. 10.1016/J.NEURON.2006.04.004. 16630825

[R93] Martin A, 2016. GRAPES—Grounding representations in action, perception, and emotion systems: How object properties and categories are represented in the human brain. Psychon. Bull. Rev. 23, 979–990. 10.3758/s13423-015-0842-3. 25968087 PMC5111803

[R94] Martin L, Durisko C, Moore MW, Coutanche MN, Chen D, Fiez JA, 2019. The VWFA is the home of orthographic learning when houses are used as letters, 17 eNeuro 6, 0425. 10.1523/ENEURO.0425-17.2019. PMC6378324 30783613

[R95] Maurer D, Ghloum JK, Gibson LC, Watson MR, Chen LM, Akinsc K, Enns JT, Hensch TK, Werker JF, 2020. Reduced perceptual narrowing in synesthesia. Proc. Natl. Acad. Sci. USA 117, 10089–10096. 10.1073/PNAS.1914668117/SUPPL_FILE/PNAS.1914668117.SAPP.PDF. 32321833 PMC7211996

[R96] Maurer D, Gibson LC, Spector F, 2012. Infant synaesthesia: New insights into the development of multisensory perception. Multisens. Dev. 10.1093/ACPROF:OSO/9780199586059.003.0010.

[R97] Maurer D, Le Grand R, Mondloch CJ, 2002. The many faces of configural processing. Trends Cogn. Sci. 6, 255–260. 10.1016/S1364-6613(02)01903-4. 12039607

[R98] Mccarthy G, Blamire AM, Rothman DL, Gruetter R, Shulman RG, 1993. Echo-planar magnetic resonance imaging studies of frontal cortex activation during word generation in humans. Proc. Natl. Acad. Sci. USA 90, 4952–4956. 10.1073/pnas.90.11.4952. 8506340 PMC46631

[R99] Mesulam MM, Wieneke C, Hurley R, Rademaker A, Thompson CK, Weintraub S, Rogalski EJ, 2013. Words and objects at the tip of the left temporal lobe in primary progressive aphasia. Brain 136, 601–618. 10.1093/BRAIN/AWS336. 23361063 PMC3572925

[R100] Michon M, López V, Aboitiz F, 2019. Origin and evolution of human speech: Emergence from a trimodal auditory, visual and vocal network. Prog. Brain Res. 250, 345–371. 10.1016/bs.pbr.2019.01.005. 31703907

[R101] Moore MW, Durisko C, Perfetti CA, Fiez JA, 2014. Learning to read an alphabet of human faces produces left-lateralized training effects in the fusiform gyrus. J. Cogn. Neurosci. 26, 896–913. 10.1162/JOCN_A_00506. 24168219 PMC4134934

[R102] Müller F, Niso G, Samiee S, Ptito M, Baillet S, Kupers R, 2019. A thalamocortical pathway for fast rerouting of tactile information to occipital cortex in congenital blindness. Nat. Commun. 10, 5154. 10.1038/s41467-019-13173-7. 31727882 PMC6856176

[R103] Nordt M, Gomez J, Natu VS, Rezai AA, Finzi D, Kular H, Grill-Spector K, 2021. Cortical recycling in high-level visual cortex during childhood development. Nat. Hum. Behav. 5, 1686–1697. 10.1038/s41562-021-01141-5. 34140657 PMC8678383

[R104] Op de Beeck HP, Pillet I, Ritchie JB, 2019. Factors determining where category selective areas emerge in visual cortex. Trends Cogn. Sci. 23, 784–797. 10.1016/j.tics.2019.06.006. 31327671

[R105] Pan Y, Popov T, Frisson S, Jensen O, 2023. Saccades are locked to the phase of alpha oscillations during natural reading. PLoS Biol. 21, e3001968. 10.1371/journal.pbio.3001968. 36649331 PMC9882905

[R106] Patterson K, Lambon Ralph MA, 1999. Selective disorders of reading? Curr. Opin. Neurobiol. 9, 235–239. 10.1016/S0959-4388(99)80033-6. 10322178

[R107] Petersen SE, Fox PT, Posner MI, Mintun M, Raichle ME, 1988. Positron emission tomographic studies of the cortical anatomy of single-word processing. Nature 331, 585–589. 10.1038/331585a0. 3277066

[R108] Plaut DC, Behrmann M, 2011. Complementary neural representations for faces and words: a computational exploration. Cogn. Neuropsychol. 28, 251–275. 10.1080/02643294.2011.609812. 22185237

[R109] Plaut DC, McClelland JL, Seidenberg MS, Patterson K, 1996. Understanding Normal and Impaired Word Reading: Computational Principles in Quasi-Regular Domains. Psychol. Rev. 103, 56–115. 10.1037/0033-295X.103.1.56. 8650300

[R110] Price CJ, Devlin JT, 2003. The myth of the visual word form area. NeuroImage 19, 473–481. 10.1016/S1053-8119(03)00084-3. 12880781

[R111] Price CJ, Devlin JT, 2011. The Interactive Account of ventral occipitotemporal contributions to reading. Trends Cogn. Sci. 15, 246–253. 10.1016/j.tics.2011.04.001. 21549634 PMC3223525

[R112] Ptito M, Schneider FCG, Paulson OB, Kupers R, 2008. Alterations of the visual pathways in congenital blindness. Exp. Brain Res 187, 41–49. 10.1007/s00221-008-1273-4. 18224306

[R113] Pulliam G, Feldman JI, Wallace MT, Cutting LE, Woynaroski TG, 2025. Associations Between Audiovisual Integration and Reading Comprehension in Autistic and Non-autistic School-Aged Children. J. Autism Dev. Disord. 10.1007/s10803-025-06960-3. PMC13063400 40715978

[R114] Ralph MAL, Jefferies E, Patterson K, Rogers TT, 2017. The neural and computational bases of semantic cognition. Nat. Rev. Neurosci. 18, 42–55. 10.1038/nrn.2016.150. 27881854

[R115] Ramachandran VS, Hubbard EM, 2001. Synaesthesia-A Window Into Perception, Thought and Language. J. Conscious. Stud. 8, 3–34.

[R116] Rayner K, 2009. The 35th Sir Frederick Bartlett Lecture: Eye movements and attention in reading, scene perception, and visual search. Q. J. Exp. Psychol. 62, 1457–1506. 10.1080/17470210902816461. 19449261

[R117] Reich L, Szwed M, Cohen L, Amedi A, 2011. A ventral visual stream reading center independent of visual experience. Curr. Biol. 21, 363–368. 10.1016/J.CUB.2011.01.040. 21333539

[R118] Rice GE, Caswell H, Moore P, Hoffman P, Lambon Ralph MA, 2018. The Roles of Left Versus Right Anterior Temporal Lobes in Semantic Memory: A Neuropsychological Comparison of Postsurgical Temporal Lobe Epilepsy Patients. Cereb. Cortex 28, 1487–1501. 10.1093/CERCOR/BHX362. 29351584 PMC6093325

[R119] Richlan F, Kronbichler M, Wimmer H, 2009. Functional abnormalities in the dyslexic brain: A quantitative meta-analysis of neuroimaging studies. Hum. Brain Mapp. 30, 3299–3308. 10.1002/HBM.20752. 19288465 PMC2989182

[R120] Riesenhuber M, Glezer LS, 2017. Evidence for rapid localist plasticity in the ventral visual stream: The example of words. Lang. Cogn. Neurosci. 32, 286. 10.1080/23273798.2016.1210178. 29201934 PMC5708570

[R121] Ritchie JB, Wardle SG, Vaziri-Pashkam M, Kravitz DJ, Baker CI, 2025. Rethinking category-selectivity in human visual cortex. Cogn. Neurosci. 1–28. 10.1080/17588928.2025.2543890. 40836402 PMC12458057

[R122] Rosenke M, Van Hoof R, Van Den Hurk J, Grill-Spector K, Goebel R, 2021. A Probabilistic Functional Atlas of Human Occipito-Temporal Visual Cortex. Cereb. Cortex 31, 603–619. 10.1093/CERCOR/BHAA246. 32968767 PMC7727347

[R123] Sabsevitz DS, Middlebrooks EH, Tatum W, Grewal SS, Wharen R, Ritaccio AL, 2020. Examining the function of the visual word form area with stereo EEG electrical stimulation: A case report of pure alexia. Cortex 129, 112–118. 10.1016/J.CORTEX.2020.04.012. 32442776

[R124] Saygin ZM, Osher DE, Norton ES, Youssoufian DA, Beach SD, Feather J, Gaab N, Gabrieli JDE, Kanwisher N, 2016. Connectivity precedes function in the development of the visual word form area. Nat. Neurosci. 19, 1250–1255. 10.1038/nn.4354. 27500407 PMC5003691

[R125] Seidenberg MS, 2005. Connectionist models of word reading. Curr. Dir. Psychol. Sci. 14, 238–242. 10.1111/j.0963-7214.2005.00372.x.

[R126] Seidenberg MS, McClelland JL, 1989. A Distributed, Developmental Model of Word Recognition and Naming. Psychol. Rev. 96, 523–568. 10.1037/0033-295X.96.4.523. 2798649

[R127] Shekari E, Nozari N, 2023. A narrative review of the anatomy and function of the white matter tracts in language production and comprehension. Front. Hum. Neurosci. 17, 1139292. 10.3389/fnhum.2023.1139292. 37051488 PMC10083342

[R128] Silani G, Frith U, Demonet JF, Fazio F, Perani D, Price C, Frith CD, Paulesu E, 2005. Brain abnormalities underlying altered activation in dyslexia: a voxel based morphometry study. Brain 128, 2453–2461. 10.1093/BRAIN/AWH579. 15975942

[R129] Simmons WK, Martin A, 2009. The anterior temporal lobes and the functional architecture of semantic memory. J. Int Neuropsychol. Soc. 15, 645–649. 10.1017/S1355617709990348. 19631024 PMC2791360

[R130] Siuda-Krzywicka K, Bola Ł, Paplińska M, Sumera E, Jednoróg K, Marchewka A, Śliwińska MW, Amedi A, Szwed M, 2016. Massive cortical reorganization in sighted braille readers. eLife 5, e10762. 10.7554/eLife.10762. 26976813 PMC4805536

[R131] Snyder KM, Forseth KJ, Donos C, Rollo PS, Fischer-Baum S, Breier J, Tandon N, 2023. Critical role of the ventral temporal lobe in naming. Epilepsia 64, 1200–1213. 10.1111/epi.17555. 36806185 PMC10312312

[R132] Srihasam K, Vincent JL, Livingstone MS, 2014. Novel domain formation reveals proto-architecture in inferotemporal cortex. Nat. Neurosci. 17, 1776–1783. 10.1038/nn.3855. 25362472 PMC4241119

[R133] Stevens WD, Kravitz DJ, Peng CS, Tessler MH, Martin A, 2017. Privileged functional connectivity between the visual word form area and the language system. J. Neurosci. 37, 5288–5297. 10.1523/JNEUROSCI.0138-17.2017. 28450544 PMC5456110

[R134] Szwed M, Dehaene S, Kleinschmidt A, Eger E, Valabrègue R, Amadon A, Cohen L, 2011. Specialization for written words over objects in the visual cortex. NeuroImage 56, 330–344. 10.1016/J.NEUROIMAGE.2011.01.073. 21296170

[R135] Taylor JSH, Rastle K, Davis MH, 2013. Can cognitive models explain brain activation during word and pseudoword reading? A meta-analysis of 36 neuroimaging studies. Psychol. Bull. 139, 766–791. 10.1037/A0030266. 23046391

[R136] Tomasino B, Ius T, Skrap M, Luzzatti C, 2020. Phonological and surface dyslexia in individuals with brain tumors: Performance pre-, intra-, immediately post-surgery and at follow-up. Hum. Brain Mapp. 41, 5015–5031. 10.1002/HBM.25176. 32857483 PMC7643394

[R137] Tomasino B, Marin D, Maieron M, D’Agostini S, Fabbro F, Skrap M, Luzzatti C, 2015. Double-letter processing in surface dyslexia and dysgraphia following a left temporal lesion: A multimodal neuroimaging study. Cortex 73, 112–130. 10.1016/J.CORTEX.2015.08.010. 26407482

[R138] Ueno T, Meteyard L, Hoffman P, Murayama K, 2018. The Ventral Anterior Temporal Lobe has a Necessary Role in Exception Word Reading. Cereb. Cortex 28, 3035–3045. 10.1093/CERCOR/BHY131. 29878073 PMC6041960

[R139] van Laarhoven T, Keetels M, Schakel L, Vroomen J, 2018. Audio-visual speech in noise perception in dyslexia. Dev. Sci. 21, e12504. 10.1111/DESC.12504. 27990724

[R140] Vandermosten M, Boets B, Poelmans H, Sunaert S, Wouters J, Ghesquière P, 2012a. A tractography study in dyslexia: neuroanatomic correlates of orthographic, phonological and speech processing. Brain 135, 935–948. 10.1093/BRAIN/AWR363. 22327793

[R141] Vandermosten M, Boets B, Wouters J, Ghesquière P, 2012b. A qualitative and quantitative review of diffusion tensor imaging studies in reading and dyslexia. Neurosci. Biobehav. Rev. 36, 1532–1552. 10.1016/J.NEUBIOREV.2012.04.002. 22516793

[R142] Veit MJ, Kucyi A, Hu W, Zhang C, Zhao B, Guo Z, Yang B, Sava-Segal C, Perry C, Zhang J, Zhang K, Parvizi J, 2021. Temporal order of signal propagation within and across intrinsic brain networks. Proc. Natl. Acad. Sci. USA 118, e2105031118. 10.1073/pnas.2105031118. 34819365 PMC8640784

[R143] Vinckier F, Dehaene S, Jobert A, Dubus JP, Sigman M, Cohen L, 2007. Hierarchical Coding of Letter Strings in the Ventral Stream: Dissecting the Inner Organization of the Visual Word-Form System. Neuron 55, 143–156. 10.1016/J.NEURON.2007.05.031. 17610823

[R144] Vogel AC, Petersen SE, Schlaggar BL, 2012. The left occipitotemporal cortex does not show preferential activity for words. Cereb. Cortex 22, 2715–2732. 10.1093/CERCOR/BHR295. 22235035 PMC3491762

[R145] Wandell BA, Dumoulin SO, Brewer AA, 2007. Visual Field Maps in Human Cortex. Neuron 56, 366–383. 10.1016/j.neuron.2007.10.012. 17964252

[R146] Wang L, Mruczek REB, Arcaro MJ, Kastner S, 2015. Probabilistic Maps of Visual Topography in Human Cortex. Cereb. Cortex 25, 3911–3931. 10.1093/cercor/bhu277. 25452571 PMC4585523

[R147] Warrington EK, Shallice T, 1980. Word-form dyslexia. Brain 103, 99–112. 10.1093/brain/103.1.99. 6244876

[R148] Waters D, Campbell R, Capek CM, Woll B, David AS, McGuire PK, Brammer MJ, MacSweeney M, 2007. Fingerspelling, signed language, text and picture processing in deaf native signers: The role of the mid-fusiform gyrus. NeuroImage 35, 1287–1302. 10.1016/J.NEUROIMAGE.2007.01.025. 17363278 PMC3480647

[R149] Whaley ML, Kadipasaoglu CM, Cox SJ, Tandon N, 2016. Modulation of Orthographic Decoding by Frontal Cortex. J. Neurosci. 36, 1173–1184. 10.1523/JNEUROSCI.2985-15.2016. 26818506 PMC4728723

[R150] White AL, Palmer J, Boynton GM, Yeatman JD, 2019. Parallel spatial channels converge at a bottleneck in anterior word-selective cortex. Proc. Natl. Acad. Sci. USA 116, 10087–10096. 10.1073/pnas.1822137116. 30962384 PMC6525533

[R151] Wilson SM, Brambati SM, Henry RG, Handwerker DA, Agosta F, Miller BL, Wilkins DP, Ogar JM, Gorno-Tempini ML, 2009. The neural basis of surface dyslexia in semantic dementia. Brain 132, 71–86. 10.1093/brain/awn300. 19022856 PMC2638692

[R152] Wilson MA, Joubert S, Ferré P, Belleville S, Ansaldo AI, Joanette Y, Rouleau I, Brambati SM, 2012. The role of the left anterior temporal lobe in exception word reading: Reconciling patient and neuroimaging findings. NeuroImage 60, 2000–2007. 22361167 10.1016/j.neuroimage.2012.02.009

[R153] Woollams AM, Ralph MAL, Plaut DC, Patterson K, 2007. SD-squared: On the association between semantic dementia and surface dyslexia. Psychol. Rev. 114, 316–339. 10.1037/0033-295X.114.2.316. 17500629

[R154] Woolnough O, Donos C, Curtis A, Rollo PS, Roccaforte ZJ, Dehaene S, Fischer-Baum S, Tandon N, 2022. A Spatiotemporal Map of Reading Aloud. J. Neurosci. 42, 5438–5450. 10.1523/JNEUROSCI.2324-21.2022. 35641189 PMC9270918

[R155] Woolnough O, Donos C, Rollo PS, Forseth KJ, Lakretz Y, Crone NE, Fischer-Baum S, Dehaene S, Tandon N, 2021. Spatiotemporal dynamics of orthographic and lexical processing in the ventral visual pathway. Nat. Hum. Behav. 5, 389–398. 10.1038/s41562-020-00982-w. 33257877 PMC10365894

[R156] Woolnough O, Tandon N, 2024. Dissociation of reading and naming in ventral occipitotemporal cortex. Brain 147, 2522–2529. 10.1093/brain/awae027. 38289871 PMC11224612

[R157] Yablonski M, Karipidis II, Kubota E, Yeatman JD, 2024. The transition from vision to language: Distinct patterns of functional connectivity for subregions of the visual word form area. Hum. Brain Mapp. 45, e26655. 10.1002/hbm.26655. 38488471 PMC10941549

[R158] Yang J, Zevin JD, Shu H, McCandliss BD, Li P, 2006. A “triangle model” of chinese reading. 28th Annu. Conf. Cogn. Sci. Soc. Proc. 912–917.

[R159] Yeatman JD, Dougherty RF, Rykhlevskaia E, Sherbondy AJ, Deutsch GK, Wandell BA, Ben-Shachar M, 2011. Anatomical properties of the arcuate fasciculus predict phonological and reading skills in children. J. Cogn. Neurosci. 23, 3304–3317. 10.1162/JOCN_A_00061. 21568636 PMC3214008

[R160] Yeatman JD, Rauschecker AM, Wandell BA, 2013. Anatomy of the visual word form area: adjacent cortical circuits and long-range white matter connections. Brain Lang. 125, 146–155. 10.1016/j.bandl.2012.04.010. 22632810 PMC3432298

[R161] Yeh F-C, Badre D, Verstynen T, 2016. Connectometry: A statistical approach harnessing the analytical potential of the local connectome. NeuroImage 125, 162–171. 10.1016/j.neuroimage.2015.10.053. 26499808

