# Supplementary material for: The reading brain – Transforming vision into language
Source: Prog Neurobiol. Author manuscript; Available in PMC 2026 Aug 20. (PMC13491551; doi:10.1016/j.pneurobio.2026.102894)
Supplement: Supplementary file 1 [file NIHMS2179612-supplement-1.pdf]

Peer Review Overview

Manuscript Title: "The Reading Brain – Transforming Vision into Language"

Received	02-Jun-2025
1st Decision	03-Oct-2025
Revision Submitted	28-Jan-2026
Accepted	17-Feb-2026

Decision Letter

Dear Dr Ghuman,

Thank you for submitting your manuscript to Progress in Neurobiology. We have received comments from reviewers on your manuscript. Your paper should become acceptable for publication pending suitable minor revision and modification of the article in light of the appended reviewer comments.

When resubmitting your manuscript, please carefully consider all issues mentioned in the reviewers' comments, outline every change made point by point, and provide suitable rebuttals for any comments not addressed.

To submit your revised manuscript go to https://www.editorialmanager.com/proneu/ and log in as an Author where you will see a menu item called 'Submission Needing Revision'.

Please resubmit your manuscript by **Nov 02, 2025**.

Research Elements (optional)
This journal encourages you to share research objects - including your raw data, methods, protocols, software, hardware and more – which support your original research article in a Research Elements journal. Research Elements are open access, multidisciplinary, peer-reviewed journals which make the objects associated with your research more discoverable, trustworthy and promote replicability and reproducibility. As open access journals, there may be an Article Publishing Charge if your paper is accepted for publication. Find out more about the Research Elements journals at https://www.elsevier.com/authors/tools-and-resources/research-elements-journals?dgcid=ec_em_research_elements_email.

We look forward to receiving your revised manuscript.

Kind regards,

Brett L. Foster
Associate Editor

Laura Colgin
Co-Editor-in-Chief
Progress in Neurobiology

Comments from the Editors and Reviewers:

Reviewer #1: Boring et al review recent developments in research into the VWFA. They compile results across functional neuroimaging, lesion, intracranial recording, and cortical stimulation to show a multifaceted view of the VWFA. This is an excellent and accessible primer for the current state of research in the VWFA. Below I discuss some areas of the manuscript the authors may consider updating or building upon:

Content:

Line 49: "The fact that we hear words in our heads while we read" While this is true for the majority of people this phenomenon is not universal and many people can read without 'hearing' an overt, internal phonological representation (e.g. Hinwar and Lambert (2021) Frontiers in Psychology).

"The Anatomy of the Early Visual-to-Orthographic Pathway" In this section it is hinted at but never explicitly stated that, within category selective cortex, the peripheral-to-foveal axis is typically medial-to-lateral.

Line 260: "Functional and anatomical connectivity studies implicate connections between VWFA and perisylvian language regions involved in phonological processing as the key pathway for orthographic-to-phonological mapping" I would suggest that it is 'a' key pathway rather than 'the' key pathway. Beyond perisylvian regions, orthographic-to-phonological mappings have also been thought to involve precentral gyrus/sulcus (e.g. Dickens et al (2019) Journal of Neuroscience, Tomasino et al (2020) Human Brain Mapping, Kaestner et al (2021) Neurobiology of Language, Woolnough et al (2022) Journal of Neuroscience). In general, the 'Orthographic-to-Phonological Pathway' section as a whole would benefit with greater anatomical specificity relating to distinct cortical regions, especially those implicated in acquired phonological dyslexia.

I would also recommend caution in the use of 'language regions' in this context, as in the field this can commonly evoke regions involved in higher-order language, i.e. sentence processing, which are likely distinct from the sublexical, phonological processes discussed here and throughout the manuscript.

"The Anatomy of the Orthographic-to-Lexicosemantic Pathway" The authors currently cover an in-depth view of lexicosemantics within VTC, however I would suggest including some further discussion of regions outside of VTC, such as within frontal and parietal lobe, that are implicated in lexical semantics while reading.

Line 331: "Resections of left BTLS for the treatment of epilepsy manifest with mild and inconsistent naming and word finding deficits (Rice et al., 2018), whereas bilateral BTLS degeneration associated with semantic dementia causes consistent and more severe naming deficits (Binney et al., 2016; Mesulam et al., 2013)." This can at least partially be explained by surgical practices to minimize naming deficits during resection, rather than indiscriminately lesioning the area as is the case in semantic dementia. Also, the Rice et al study compares post-resection epilepsy patients to non-epileptic controls. Better references would be Binder et al (2020) Epilepsia, Kaestner et al (2021) NeuroImage Clinical, and Snyder et al (2023) Epilepsia, who use pre-post resection data that show substantial naming deficits in left hemisphere resections, but only once the resection continues far enough posteriorly from the temporal pole.

Line 405: "Alternatively, VWFA activation in individuals who are blind may occur via bottom-up projections from the ventral stream, as it does in sighted individuals." It's unclear how this would be possible. Activation in early visual areas during visual imagery in sighted individuals is typically as a result of a top-down anterior-to-posterior spread of activation and this appears to be the likely source of activation for blind individuals as well.

Formatting/Text:

Typo, Line 126: "It also has connectivity into a ventral-to-anterior temporal and frontal cortex pathway maps between visual forms and their names and meaning,"

Typo, Line 253: "EG visual categories"

Formatting error, Line 312: (Figure 2AError! Reference source not found.)

Typo, Line 547: "though becoming a letter-by-letter reading following acquired alexia after damage to VWFA"

Figure 4 legend: "Electrical brain stimulation across left VTC of 41 patients" 49 patients

Reviewer #2: Thank you for the opportunity to review this excellent manuscript. The authors present a thorough and compelling review of theoretical frameworks and neurobiological evidence surrounding the brain's reading circuit, with a central focus on the visual word form area (VWFA). The manuscript synthesizes strong support for the ideas that "large-scale brain connectivity drives specificity of local neural representations" and that "preexisting anatomical pathways constrain VWFA's localization" in service of reading-related processes.

The manuscript does an outstanding job of integrating prior literature. It begins with historical perspectives on the VWFA's relationship to reading, bridges theoretical frameworks of reading-related processes, and presents neuroimaging evidence that maps intersecting white matter pathways to these processes. The authors bring together findings across multiple modalities, including functional MRI (BOLD activation during reading tasks), functional connectivity analyses, diffusion MRI of structural connectivity, and brain stimulation studies, while also incorporating evidence from clinical populations. Together, this evidence provides a strong foundation for the bidirectionality hypothesis of VWFA connectivity: that the VWFA does not simply "send" visual word forms forward, but that its functional tuning and role in reading depend critically on feedback from connected regions.

The manuscript also highlights "a model in which the VWFA serves as a key hub for orthographic processing by acting as a waypoint through which lower-level visual centers can access visuo-linguistic circuitry established prior to reading." This review was a pleasure to read, and with minor revisions, I believe it will be ready for publication.

//

Section 1: History and Overview
Section 2: Cognitive Models of Reading
Section 3: The Organization of VWFA and Its Representations

The authors provide excellent historical context, tracing early debates on the VWFA's role in reading. They describe key processes proposed by prior models, such as orthography-to-phonology and orthography-to-semantics mappings. These frameworks explain not only processes in skilled reading but also disruptions in developmental and acquired dyslexia.

The authors also present a neurobiological framework that links white matter pathways intersecting the VWFA to core reading processes:
- Ventral visual pathway (via the VOF) supporting recognition of letters and words.
- Ventral-to-lateral temporal pathway (via the AF) supporting visual-to-phonological transformations.
- Ventral-to-anterior temporal and frontal pathways (via the ILF/IFOF) supporting visual-to-semantic mappings.

This evidence converges on the hypothesis that large-scale connectivity shapes local VWFA representations.

The discussion of theoretical frameworks—including the Dual Route Model (direct orthographic-lexicosemantic vs. indirect orthographic-phonological routes) and the Triangle Model (adaptive parallel-distributed networks of phonological, orthographic, and semantic systems)—was clear and well-situated.

Figure 2 is a particular strength. it elegantly illustrates how white matter pathways intersecting with the VWFA support reading processes. The VOF supports word-form and visual processing, the AF links word forms with phonology, and the ILF/IFOF link word forms with semantics.

//

Section 4: Preexisting Anatomical Pathways Constrain VWFA's Localization
Sections 5-7: Early Visual-to-Orthographic, Orthographic-to-Phonological, Orthographic-to-Lexicosemantic Pathways

The authors do an excellent job reviewing the VWFA's connectivity with white matter pathways that support reading. The "Orthographic-to-Lexicosemantic Pathway" section is particularly robust, linking

VWFA function to the basal temporal language system (BTLS) through ILF/IFOF connectivity. Similarly, the "Orthographic-to-Phonological Pathway" is clearly articulated through the AF.

By contrast, the "Early Visual-to-Orthographic Pathway" section could be further strengthened by following the conceptual clarity and logic used in the other two sections. This would help balance the manuscript's three core organizing sections.

Suggestions for strengthening these sections:
- Integration with Figure 2: While Figure 2 and its caption provide excellent tract descriptions, incorporating these details directly into the relevant sections would make the narrative more cohesive. This is particularly important for the Early Visual-to-Orthographic Pathway (and the VOF).
- Terminal regions and systems: Consider describing the endpoints of each tract, the cognitive systems they subserve, and how they contribute to VWFA's reading role. For example:
o Early Visual-to-Orthographic: VOF connecting VWFA with dorsal visual and parietal attention regions, supporting eye-movement coordination.
o Orthographic-to-Phonological: AF connecting VWFA to frontal/temporal speech and motor regions, supporting orthography-to-phonology conversion.
o Orthographic-to-Lexicosemantic: ILF/IFOF connecting VWFA to anterior temporal and frontal semantic regions, supporting lexical-semantic processes.
- Multi-modal evidence: Strengthen each section by pairing structural connectivity evidence (diffusion MRI) with functional evidence (fMRI, clinical cases, stimulation studies).
- Reorganizing Section 9: Integrating "Reading and Eye Movements" with Section 5 (Orthographic-to-Phonological Pathway) could strengthen the account of the VOF and attentional/eye-movement processes.
- Bidirectionality: Since the manuscript emphasizes bidirectionality, consider adding clinical and stimulation evidence showing how disruption of tract endpoints alters reading processes.
- Link to classical models: It would also enrich the manuscript to situate the Orthographic-to-Phonological and Orthographic-to-Lexicosemantic pathways within classical neurobiological models of speech and language (e.g., Hickok & Poeppel, 2007; Friederici, 2011; Shekari & Nozari, 2023).

The mention of audiovisual integration, such as lip-reading and its overlap with word-reading pathways, is particularly intriguing. Recent work in autism (e.g., Pulliam et al., 2025) may provide valuable insights into these integrative processes and could be briefly discussed.

//

Section 11: Extant Models of VWFA
Section 12: Concluding Remarks

The authors provide a balanced account of extant models of VWFA and ongoing debates about its location and function. Their emphasis on adaptation of preexisting circuits for naming, semantics, and phonology, combined with the importance of both top-down and bottom-up interactions, underscores the central theme of bidirectional connectivity.

//

This manuscript is a comprehensive, well-structured, and engaging review that integrates theoretical frameworks, multimodal neuroimaging, and clinical evidence. My suggestions primarily concern strengthening balance across sections, integrating figure details, and expanding on endpoint regions and classical models. With these refinements, this manuscript will make a strong and impactful contribution to the literature. Thank you again for the opportunity, and I look forward to reading the revised submission.

References for neurobiological models of speech and language to incorporate/discuss:
Hickok, G., & Poeppel, D. (2007). The cortical organization of speech processing. Nature reviews neuroscience, 8(5), 393-402.
Friederici, A. D. (2011). The brain basis of language processing: from structure to function. Physiological reviews, 91(4), 1357-1392.
Shekari, E., & Nozari, N. (2023). A narrative review of the anatomy and function of the white matter tracts in language production and comprehension. Frontiers in Human Neuroscience, 17, 1139292.

Research on language system, VWFA, and white matter pathway to incorporate/discuss:
Fan, Q., Anderson, A. W., Davis, N., & Cutting, L. E. (2014). Structural connectivity patterns associated with the putative visual word form area and children's reading ability. Brain research, 1586, 118-129.
Yablonski, M., Karipidis, I. I., Kubota, E., & Yeatman, J. D. (2024). The transition from vision to

language: Distinct patterns of functional connectivity for subregions of the visual word form area. Human Brain Mapping, 45(4), e26655.

Research on audiovisual integration and reading in clinical populations to incorporate/discuss:
Pulliam, G., Feldman, J. I., Wallace, M. T., Cutting, L. E., & Woynaroski, T. G. (2025). Associations Between Audiovisual Integration and Reading Comprehension in Autistic and Non-autistic School-Aged Children. Journal of Autism and Developmental Disorders, 1-13.

We would like to sincerely thank the reviewers for their comments on our manuscript, “The Reading Brain – Transforming Vision into Language”. We appreciate the reviewers’ enthusiasm for this work. We greatly appreciate their very thoughtful comments and believe that the manuscript is greatly improved by responding to them.

Reviewer #1: Boring et al review recent developments in research into the VWFA. They compile results across functional neuroimaging, lesion, intracranial recording, and cortical stimulation to show a multifaceted view of the VWFA. This is an excellent and accessible primer for the current state of research in the VWFA. Below I discuss some areas of the manuscript the authors may consider updating or building upon:

Content:

Line 49: "The fact that we hear words in our heads while we read" While this is true for the majority of people this phenomenon is not universal and many people can read without 'hearing' an overt, internal phonological representation (e.g. Hinwar and Lambert (2021) Frontiers in Psychology).

Thank you for pointing this out. Indeed, in our opinion, one area of the science the neurobiology of reading that is ripe for additional study is to understand how the neurobiology differs in those who read without hearing words in their heads (similarly those who are congenitally deaf). We have thus updated this statement to read “The fact that most people hear words in our heads...” It is notable though that historically, the debate does arise from this point as hearing words in one’s mind is the central reasoning that Wernicke used to exclude the notion of a visual center for words in the brain and reject Dejerine’s model of reading.

We have also added this point regarding those who do not hear words in their heads and the congenitally deaf to the outstanding questions box at the end of the review as an open question in the neurobiology of reading.

"The Anatomy of the Early Visual-to-Orthographic Pathway" In this section it is hinted at but never explicitly stated that, within category selective cortex, the peripheral-to-foveal axis is typically medial-to-lateral.

Thank you for pointing this out, we have added this point into that section explicitly.

Line 260: "Functional and anatomical connectivity studies implicate connections between VWFA and perisylvian language regions involved in phonological processing as the key pathway for orthographic-to-phonological mapping" I would suggest that it is 'a' key pathway rather than 'the' key pathway. Beyond perisylvian regions, orthographic-to-phonological mappings have also been thought to involve precentral gyrus/sulcus (e.g. Dickens et al (2019) Journal of Neuroscience, Tomasino et al (2020) Human Brain Mapping, Kaestner et al (2021) Neurobiology of Language, Woolnough et al (2022) Journal of Neuroscience). In general, the 'Orthographic-to-Phonological Pathway' section as a whole would benefit with greater anatomical specificity relating to distinct cortical regions, especially those implicated in acquired phonological dyslexia.

Thank you for this comment and these points. “Perisylvian” in this case is primarily meant as a catch-all term for the areas that are served by the arcuate fasciculus, and thus not meant to exclude the precentral gyrus/sulcus. We have added the following text to clarify this point:

Note that we use “perisylvian language areas” loosely to refer to the phonological and articulatory processing areas served by the arcuate fasciculus and thus includes frontal areas somewhat more dorsal than are typically described as perisylvian that have sometimes been implicated in phonological and subvocal articulatory processing in reading (Tomasino et al., 2020; Woolnough et al., 2022).

Also note that in response to suggestions from reviewer 2, we have further described this pathway as the one that connects the VWFA to temporal and frontal regions involved in phonological processing and articulatory planning. We hope these more in depth descriptions help clarify that we did not mean to exclude regions that connect to the arcuate that are not strictly perisylvian.

I would also recommend caution in the use of 'language regions' in this context, as in the field this can commonly evoke regions involved in higher-order language, i.e. sentence processing, which are likely distinct from the sublexical, phonological processes discussed here and throughout the manuscript.

We hope that with the more extensive and clear definitions of these regions, that it should be clear what we are referring to. In particular, based on your suggestions and suggestions from reviewer 2, we are clearer that these are pathways that have been implicated in phonological and prearticulatory processing. This is ultimately a term used because it is relatively common in the field for these regions that sit along the arcuate fasciculus as the "language loop."

"The Anatomy of the Orthographic-to-Lexicosemantic Pathway" The authors currently cover an in-depth view of lexicosemantics within VTC, however I would suggest including some further discussion of regions outside of VTC, such as within frontal and parietal lobe, that are implicated in lexical semantics while reading.

We now realize that in our prior version of the manuscript, we had an insufficient description of how the IFOF and ILF continued particularly into frontal areas involved in lexicosemantic processing. In the figure legend of figure 2, it mentions:

The inferior frontooccipital fasciculus (IFOF, green) and inferior longitudinal fasciculus (ILF, pink) connects VWFA with anterior regions of ventral temporal cortex and frontal lobe, which likely play important roles in the lexicosemantic pathway.

We have also now added a discussion in the text regarding these pathways:

This pathway connects the VWFA anteriorly along VTC through the temporal pole and into inferior frontal cortex that have been implicated in semantics during reading, naming, and perceptual recognition (Forseth et al., 2018; Ghuman et al., 2008; Graves et al., 2007; Luders et al., 1991; Mesulam et al., 2013).

Line 331: "Resections of left BTLS for the treatment of epilepsy manifest with mild and inconsistent naming and word finding deficits (Rice et al., 2018), whereas bilateral BTLS degeneration associated with semantic dementia causes consistent and more severe naming deficits (Binney et al., 2016; Mesulam et al., 2013)." This can at least partially be explained by surgical practices to minimize naming deficits during resection, rather than indiscriminately lesioning the area as is the case in semantic dementia. Also, the Rice et al study compares post-resection epilepsy patients to non-epileptic controls. Better

references would be Binder et al (2020) Epilepsia, Kaestner et al (2021) NeuroImage Clinical, and Snyder et al (2023) Epilepsia, who use pre-post resection data that show substantial naming deficits in left hemisphere resections, but only once the resection continues far enough posteriorly from the temporal pole.

Thank you for this comment, there is certainly important nuance here. It is perhaps more correct to say that the semantic pathway is relatively bilateral and there is some question as to how lateralized naming deficits with BTLS damage. We still feel that the bilaterality of the semantic pathway may relate to why individuals who read non-alphabetic writing systems display a more bilateral reading system. We have updated this passage to reflect critical nuances and ongoing debates with regards to naming, semantics, and lateralization:

One point to note is that, though BTLS has a substantial left hemisphere bias, aspects of the BTLS appears to be more bilaterally organized compared to the stricter left lateralization of much of the speech-language system. Resections of left BTLS for the treatment of epilepsy manifest with mild and inconsistent naming and word finding deficits (Rice et al., 2018), though others have found that larger resections of BTLS do cause naming deficits (Kaestner et al., 2022; Snyder et al., 2023). Bilateral BTLS degeneration associated with semantic dementia causes consistent and more severe naming deficits (Binney et al., 2016; Mesulam et al., 2013). Furthermore, both left and right BTLS display word selectivity at 300-400 ms after word presentation, with left VWFA becoming word selective about 100 ms earlier (Boring et al., 2021) (Figure 4A). One potential lateral asymmetry of BTLS may be between naming and semantic processing, where left BTLS is more important for naming than right BTLS, but broader semantic processing is distributed more bilaterally. Disentangling this lateralization requires examining the effects of left and right BTLS stimulation and damage on naming, verbal and non-verbal tests of semantic knowledge.

Line 405: "Alternatively, VWFA activation in individuals who are blind may occur via bottom-up projections from the ventral stream, as it does in sighted individuals." It's unclear how this would be possible. Activation in early visual areas during visual imagery in sighted individuals is typically as a result of a top-down anterior-to-posterior spread of activation and this appears to be the likely source of activation for blind individuals as well.

Thank you for this comment as we see that this point was confusingly presented and we have substantially modified how we describe this. For individuals with acquired blindness, our argument is in agreement with the reviewer's assessment of how the "visual" information

reaches visual cortex through imagery (this does not necessarily need to be V1, but could be an earlier part of the ventral visual stream than the VWFA). To reach VWFA "visual" information could then flow from visual cortex to VWFA (e.g. through the typical "bottom-up" pathway), that is what we meant by "bottom-up projections from the ventral stream, as it does in sighted individuals." However, we see that this is confusing as this remains a "top-down" process overall, though arguably it may be bottom-up from VWFA's perspective. Given this confusion and that it is not critical to the thrust of this review, we have moved the imagery section to the top-down part of this section.

Formatting/Text:

Typo, Line 126: "It also has connectivity into a ventral-to-anterior temporal and frontal cortex pathway maps between visual forms and their names and meaning,"

Typo, Line 253: "EG visual categories"

Formatting error, Line 312: (Figure 2AError! Reference source not found.)

Typo, Line 547: "though becoming a letter-by-letter reading following acquired alexia after damage to VWFA"

Figure 4 legend: "Electrical brain stimulation across left VTC of 41 patients" 49 patients

Thank you very much for catching these formatting/text issues. We have corrected them.

Reviewer #2: Thank you for the opportunity to review this excellent manuscript. The authors present a thorough and compelling review of theoretical frameworks and neurobiological evidence surrounding the brain's reading circuit, with a central focus on the visual word form area (VWFA). The manuscript synthesizes strong support for the ideas that "large-scale brain connectivity drives specificity of local neural representations" and that "preexisting anatomical pathways constrain VWFA's localization" in service of reading-related processes.

The manuscript does an outstanding job of integrating prior literature. It begins with historical perspectives on the VWFA's relationship to reading, bridges theoretical frameworks of reading-related processes, and presents neuroimaging evidence that maps intersecting white matter pathways to these processes. The authors bring together findings across multiple modalities, including functional MRI (BOLD activation during reading tasks), functional connectivity analyses, diffusion MRI of structural connectivity, and brain stimulation studies, while also incorporating evidence from clinical populations. Together, this evidence provides a strong foundation for the bidirectionality hypothesis of VWFA connectivity: that the VWFA does not simply "send" visual word forms forward, but that its functional tuning and role in reading depend critically on feedback from connected regions.

The manuscript also highlights "a model in which the VWFA serves as a key hub for orthographic processing by acting as a waypoint through which lower-level visual centers can access visuo-linguistic circuitry established prior to reading." This review was a pleasure to read, and with minor revisions, I believe it will be ready for publication.

//

Section 1: History and Overview
Section 2: Cognitive Models of Reading
Section 3: The Organization of VWFA and Its Representations

The authors provide excellent historical context, tracing early debates on the VWFA's role in reading. They describe key processes proposed by prior models, such as orthography-to-phonology and orthography-to-semantics mappings. These frameworks explain not only processes in skilled reading but also disruptions in developmental and acquired dyslexia.

The authors also present a neurobiological framework that links white matter pathways intersecting the VWFA to core reading processes:
- Ventral visual pathway (via the VOF) supporting recognition of letters and words.
- Ventral-to-lateral temporal pathway (via the AF) supporting visual-to-phonological transformations.
- Ventral-to-anterior temporal and frontal pathways (via the ILF/IFOF) supporting visual-to-semantic mappings.

This evidence converges on the hypothesis that large-scale connectivity shapes local VWFA representations.

The discussion of theoretical frameworks—including the Dual Route Model (direct orthographic-lexicosemantic vs. indirect orthographic-phonological routes) and the Triangle Model (adaptive parallel-distributed networks of phonological, orthographic, and semantic systems)—was clear and well-situated.

Figure 2 is a particular strength. it elegantly illustrates how white matter pathways intersecting with the VWFA support reading processes. The VOF supports word-form and visual processing, the AF links word forms with phonology, and the ILF/IFOF link word forms with semantics.

//

Section 4: Preexisting Anatomical Pathways Constrain VWFA's Localization
Sections 5-7: Early Visual-to-Orthographic, Orthographic-to-Phonological, Orthographic-to-Lexicosemantic Pathways

The authors do an excellent job reviewing the VWFA's connectivity with white matter pathways that support reading. The "Orthographic-to-Lexicosemantic Pathway" section is particularly robust, linking VWFA function to the basal temporal language system (BTLS) through ILF/IFOF connectivity. Similarly, the "Orthographic-to-Phonological Pathway" is clearly articulated through the AF.

By contrast, the "Early Visual-to-Orthographic Pathway" section could be further strengthened by following the conceptual clarity and logic used in the other two sections. This would help balance the manuscript's three core organizing sections.

We very much appreciate these suggestions and feel that addressing them have strengthened our work. Our response below is somewhat out of order from your original review as we grouped our responses by sections in the manuscript.

Suggestions for strengthening these sections:
- Integration with Figure 2: While Figure 2 and its caption provide excellent tract descriptions, incorporating these details directly into the relevant sections would make the narrative more cohesive. This is particularly important for the Early Visual-to-Orthographic Pathway (and the VOF).

o Early Visual-to-Orthographic: VOF connecting VWFA with dorsal visual and parietal attention regions, supporting eye-movement coordination.

- Reorganizing Section 9: Integrating "Reading and Eye Movements" with Section 5 (Visual-to-Orthographic Pathway) could strengthen the account of the VOF and attentional/eye-movement processes.

We have grouped our response to these comments together as they particularly relate to the section on the VOF and the early visual-to-orthographic pathway. Thank you for these suggestions. We fully agree that this section required more fleshing out. Bringing the reading and eye movements to section 5/the early visual-to-orthographic pathway fleshes out the role of the VOF, makes this section particularly centered around this white matter pathway, parallel to the other sections, and sharpens the narrative.

- Terminal regions and systems: Consider describing the endpoints of each tract, the cognitive systems they subserve, and how they contribute to VWFA's reading role. For example:

o Orthographic-to-Phonological: AF connecting VWFA to frontal/temporal speech and motor regions, supporting orthography-to-phonology conversion.
o Orthographic-to-Lexicosemantic: ILF/IFOF connecting VWFA to anterior temporal and frontal semantic regions, supporting lexical-semantic processes.

These are great suggestions and a good way to sharpen the description of the areas that connect to the VWFA through these tracts. Along with the suggestions from reviewer 1 to clarify what regions we are talking about for these pathways; we feel that these additions provide important detail to our readers.

- Multi-modal evidence: Strengthen each section by pairing structural connectivity evidence (diffusion MRI) with functional evidence (fMRI, clinical cases, stimulation studies).

We have tried to flesh out more of this multimodal evidence where appropriate.

- Bidirectionality: Since the manuscript emphasizes bidirectionality, consider adding clinical and stimulation evidence showing how disruption of tract endpoints alters reading processes.

This issue is a bit thorny because the types of disruption that are generally studied in humans affects both the grey and white matter – electrical stimulation is believed to travel antidromically through neural circuits and resections, strokes, and tumors generally affect the local white matter along with the grey matter. Thus, it is generally challenging to disentangle what effects of disruption are localized to tract endpoints and what are due to the disconnection and disruption of the circuit. Thus, we lean towards the more conservative approach of discussing the relationship between disruptions of reading processes and different pathways and systems, rather than tract endpoints.

- Link to classical models: It would also enrich the manuscript to situate the Orthographic-to-Phonological and Orthographic-to-Lexicosemantic pathways within classical neurobiological models of speech and language (e.g., Hickok & Poeppel, 2007; Friederici, 2011; Shekari & Nozari, 2023).

This is an excellent suggestion that helps situate our manuscript with theories of speech and language. It also helps situate the VWFA as a critical entry point for orthographic information to enter these speech and language pathways. To this end, we have added the following at the bottom of page 8:

These connections place the VWFA at an entry point for visual information to proceed into what has been called the ventral language pathway, important for lexical and semantic processing, and the dorsal language pathway, important for phonological and articulatory speech processing (Friederici, 2011; Hickok and Poeppel, 2007; Shekari and Nozari, 2023).

and added citations to these models at other places where appropriate.

The mention of audiovisual integration, such as lip-reading and its overlap with word-reading pathways, is particularly intriguing. Recent work in autism (e.g., Pulliam et al., 2025) may provide valuable insights into these integrative processes and could be briefly discussed.

Thank you very much for suggesting this reference. It fits in very nicely with our arguments and we have added the following discussion of this work to the manuscript:

A recent study demonstrated a moderate-to-large correspondence between the magnitude of the McGurk effect and reading decoding and comprehension (Pulliam et al., 2025), supporting the hypothesized shared pathway for lip/speech reading and word reading.

//

Section 11: Extant Models of VWFA
Section 12: Concluding Remarks

The authors provide a balanced account of extant models of VWFA and ongoing debates about its location and function. Their emphasis on adaptation of preexisting circuits for naming, semantics, and phonology, combined with the importance of both top-down and bottom-up interactions, underscores the central theme of bidirectional connectivity.

//

This manuscript is a comprehensive, well-structured, and engaging review that integrates theoretical frameworks, multimodal neuroimaging, and clinical evidence. My suggestions primarily concern strengthening balance across sections, integrating figure details, and expanding on endpoint regions and classical models. With these refinements, this manuscript will make a strong and impactful contribution to the literature. Thank you again for the opportunity, and I look forward to reading the revised submission.

References for neurobiological models of speech and language to incorporate/discuss:
Hickok, G., & Poeppel, D. (2007). The cortical organization of speech processing. Nature reviews neuroscience, 8(5), 393-402.
Friederici, A. D. (2011). The brain basis of language processing: from structure to function. Physiological reviews, 91(4), 1357-1392.
Shekari, E., & Nozari, N. (2023). A narrative review of the anatomy and function of the white matter tracts in language production and comprehension. Frontiers in Human Neuroscience, 17, 1139292.

Research on language system, VWFA, and white matter pathway to incorporate/discuss:
Fan, Q., Anderson, A. W., Davis, N., & Cutting, L. E. (2014). Structural connectivity patterns associated with the putative visual word form area and children′ s reading ability. Brain

research, 1586, 118-129.
Yablonski, M., Karipidis, I. I., Kubota, E., & Yeatman, J. D. (2024). The transition from vision to language: Distinct patterns of functional connectivity for subregions of the visual word form area. Human Brain Mapping, 45(4), e26655.

Research on audiovisual integration and reading in clinical populations to incorporate/discuss:

Pulliam, G., Feldman, J. I., Wallace, M. T., Cutting, L. E., & Woynaroski, T. G. (2025). Associations Between Audiovisual Integration and Reading Comprehension in Autistic and Non-autistic School-Aged Children. Journal of Autism and Developmental Disorders, 1-13.

Thank you very much for these suggested references.
